# An Abelisauroid Theropod Dinosaur from the Turonian of Madagascar

**DOI:** 10.1371/journal.pone.0062047

**Published:** 2013-04-18

**Authors:** Andrew A. Farke, Joseph J. W. Sertich

**Affiliations:** 1 Raymond M. Alf Museum of Paleontology, Claremont, California, United States of America; 2 Department of Earth Sciences, Denver Museum of Nature and Science, Denver, Colorado, United States of America; Ludwig-Maximilians-Universität München, Germany

## Abstract

Geophysical evidence strongly supports the complete isolation of India and Madagascar (Indo-Madagascar) by *∼*100 million years ago, though sparse terrestrial fossil records from these regions prior to *∼*70 million years ago have limited insights into their biogeographic history during the Cretaceous. A new theropod dinosaur, *Dahalokely tokana*, from Turonian-aged (*∼*90 million years old) strata of northernmost Madagascar is represented by a partial axial column. Autapomorphies include a prominently convex prezygoepipophyseal lamina on cervical vertebrae and a divided infraprezygapophyseal fossa through the mid-dorsal region, among others. Phylogenetic analysis definitively recovers the species as an abelisauroid theropod and weakly as a noasaurid. *Dahalokely* is the only known dinosaur from the interval during which Indo-Madagascar likely existed as a distinct landmass, but more complete material is needed to evaluate whether or not it is more closely related to later abelisauroids of Indo-Madagascar or those known elsewhere in Gondwana.

## Introduction

Since its separation from India and subsequent isolation *∼*88 million years ago (Ma) [1], [2], Madagascar has hosted a highly endemic fauna and flora. The non-marine Cretaceous faunal assemblage of Madagascar is best represented by the Maastrichtian-aged (*∼*70 million years old) Maevarano Formation, with an exquisitely-preserved assemblage of non-avian dinosaurs (including titanosaurian sauropods and noasaurid, abelisaurid, and dromaeosaurid theropods), birds, crocodyliforms, turtles, fish, mammals and frogs [3]. Many of these Maevarano Formation vertebrates have been instrumental in the formulation of hypotheses concerning the Cretaceous biogeography of Madagascar. Some initial phylogenetic results for Maevarano Formation taxa suggested a close biogeographic relationship between South America and Madagascar, with organisms potentially dispersing via a late-surviving subaerial route that included Antarctica [4]–[7]. However, further consideration of geological and palaeontological evidence does not support this dispersal hypothesis. Instead, emerging evidence suggests complete isolation of the Indo-Madagascar landmass by 100 Ma, later followed by their separation from each other *∼*88 Ma [8]–[10]. Reconstructed ghost lineages for major clades of obligate terrestrial vertebrates imply that the ancestors of the Maastrichtian fauna likely inhabited Indo-Madagascar by 100 Ma [10]. Nonetheless, confirmation of this has been greatly hampered by the paucity of pre-Maastrichtian terrestrial fossils from the Cretaceous of both the Indian subcontinent (for which only fragmentary Coniacian-aged, 89.8–86.3 Ma, and Cenomanian–Turonian, 100.5–89.8 Ma, dinosaurs are known [11]–[13]) and Madagascar (for which only isolated, undescribed elements are known from the uncertainly dated [Coniacian–?Santonian] Ankazamihaboka sandstone and nondiagnostic fragments from the Cenomanian, 100.5–93.9 million years old; [14], [15]).

Here we describe a new abelisauroid theropod, *Dahalokely tokana*, which at *∼*90 Ma is the oldest identifiable dinosaur currently known from the Cretaceous of Madagascar and potentially predates the breakup of Indo-Madagascar. Abelisauroids are carnivorous theropod dinosaurs best-represented from Gondwana, originating in the mid-Jurassic at latest and persisting to the terminal Cretaceous [16], [17]. Two main clades are recognized: noasaurids are typically small-bodied (<3 m total body length) and at least some species had unusually procumbent teeth [18]; abelisaurids are larger bodied (>4 m total body length) and often characterized by greatly reduced forelimbs and cranial ornamentation [19]–[21]. *Dahalokely tokana* is a mid-sized taxon (∼3.5 m estimated body length) at least 20 million years older than the abelisaurid *Majungasaurus crenatissimus* and the noasaurid *Masiakasaurus knopfleri* from the Maevarano Formation of northwestern Madagascar. Thus, *D. tokana* pushes back the diagnostic record of abelisauroids on the island.

## Methods

### Nomenclatural acts

The electronic edition of this article conforms to the requirements of the amended International Code of Zoological Nomenclature, and hence the new names contained herein are available under that Code from the electronic edition of this article. This published work and the nomenclatural acts it contains have been registered in ZooBank, the online registration system for the ICZN. The ZooBank LSIDs (Life Science Identifiers) can be resolved and the associated information viewed through any standard web browser by appending the LSID to the prefix “http://zoobank.org/”. The LSID for this publication is: urn:lsid:zoobank.org:pub:AA81239E-D9E9-4C5F-BBC1-567EFF984DE0. The electronic edition of this work was published in a journal with an ISSN, and has been archived and is available from the following digital repositories: PubMed Central and LOCKSS.

### Permits

All necessary permits were obtained for the described study, which complied with all relevant regulations. Fieldwork was conducted under permit from Ministère des Mines de Madagascar and in collaboration with the Ministère de l’Enseignement Supérieur et de la Recherche Scientifique de Madagascar.

### Fieldwork and preparation

**Figure 1:**
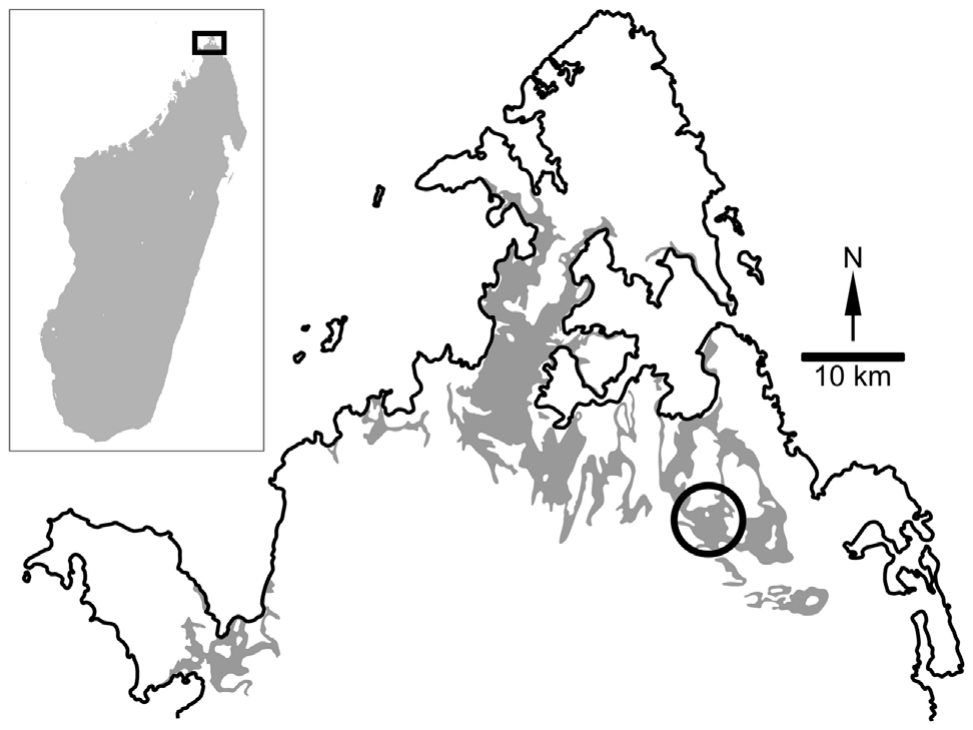
Map of the *Dahalokely tokana* holotype locality. Schematic geological map of a portion of the Diego Basin in northern Madagascar, with outcrops of sedimentary rocks of medial and Late Cretaceous age shown in gray. The general locality for the holotype of *Dahalokely tokana* (UA 9855) is indicated by the area within the circle. The inset map shows the location of the region within Madagascar. Modified after Rerat [50], de Saint Ours and Rerat [51], and de Saint Ours et al. [52].

The specimen was discovered *in situ* in the field in northernmost Madagascar (Figure 1) during the 2007 field season. Fossils were manually excavated using standard paleontological techniques (hammer and chisel, etc.), stabilized in the field using Acrysol WS-24 colloidally dispersed in water, and encased in plaster field jackets. Preparation, including additional stabilization and removal of matrix from the specimens, was completed at the Stony Brook University Vertebrate Fossil Preparation Laboratory using steel and carbide picks, pneumatic tools, and paint brushes. Additional stabilization and repair in the lab required Paraloid B-72 acrylic co-polymer dissolved in acetone. Specimens were molded using a room-temperature vulcanizing tin-cured silicone rubber (Silicones, Inc. GI-1000) and cast with a pigmented and talced wax-free polyester resin. Mold parts were defined using plastalina modeling clay and also as crack and gap filler within specimens. Specimen surfaces and mold parts were sealed with Vinac B-15 dissolved in acetone, with subsequent polymer removed through acetone dilution and wicking of polymer and acetone onto lens paper. The specimen is accessioned into the permanent collection at the University of Antananarivo (UA; Antananarivo, Madagascar). Casts were deposited at the Raymond M. Alf Museum of Paleontology (RAM, Claremont, California, USA) under specimen number RAM 16010, and the molds archived at Stony Brook University Vertebrate Fossil Preparation Laboratory (Stony Brook, New York, USA). In order to visualize and evaluate field jacket contents prior to mechanical preparation, to examine internal structures, and for the purposes of producing a digital archive, selected elements were scanned using computed tomography (CT) at Stony Brook University Hospital (Stony Brook, New York, USA). Scan parameters varied depending upon the specimen. Resulting reconstructions of selected elements are in Figures S1, S2, S3, S4, and S5.

### Measurements

Dimensions of the *Dahalokely tokana* holotype, UA 8678, were acquired to the nearest 0.1 mm using a digital calipers (Tables 1 and 2). Reference points and abbreviations follow those used by O’Connor ([22]:table 1) and are repeated here for ease of comparison.

**Table 1:** Selected measurements in millimeters of the vertebrae from the *Dahalokely tokana* holotype, UA 8678.

Vertebra	CENL	CDCW	CDCH	MIDW	TOVH	NSH	NSL	NSW	IZW	IZL	IPPW	IDPW	PP/DP	EPL
**C?5**	64.5	41.5	23.8	32.3	74.2	30.7	25.3	—	75.3	81.6	42.4	†	†	20.8
**D?1**	44.0	48.5	39.8	30.6	108.6	57.2	9.6	17.5	57.8	54.5	64.5	†	†	—
**D?2**	48.7	50.4	38.4	30.0	97.0	50.6	*21.1	16.0	50.9	56.9	60.1	158.4	0.379	—
**D?6**	52.1	46.1	41.5	21.3	121.2	65.9	28.8	11.9	26.9	76.7	94.9	136.2	0.697	—
**D?7**	55.1	46.9	42.3	22.2	116.1	62.7	34.5	11.9	27.0	83.3	101.8	126.1	0.807	—
**D?8**	55.2	47.3	41.9	22.3	120.4	66.0	32.2	11.6	27.6	76.7	108.8	134.9	0.807	—
**D?9**	†	52.9	45.4	†	126.7	69.9	33.7	10.9	24.0	†	105.6	129.7	0.814	—

**Abbreviations** (modified after [22]): CENL, maximum craniocaudal length of centrum; CDCW, maximum width of caudal articular facet of centrum; CDCH, maximum height of caudal articular facet of centrum; MIDW, width of centrum at mid-length; TOVH, total vertebral height at maximum dorsoventral extent including centrum and neural spine; NSH, maximum height of neural spine measured from dorsal surface of neural canal; NSL, craniocaudal length of neural spine at spine mid-height; NSW, transverse extent (width) of neural spine at spine mid-height; IZW, interzygapophyseal width, the distance between lateral margins of postzygapophyses; IZL, interzygapophyseal length, the distance from cranial margin of right prezygapophysis to caudal margin of right postzygapophysis; IPPW, interparapophyseal width, measured between lateral limits of parapophyses; IDPW, interdiapophyseal width, measured between lateral limits of diapophyses; PP/DP, Para-Diapophyseal Index, ratio of IPPW to IDPW; EPL, epipophyseal length, measured from caudal margin of postzygapophyseal facet to caudalmost extent of epipophysis. —, measurement not applicable for element; †, element incomplete for measurement. *excludes ossified interspinous ligament.

**Table 2:** Selected measurements in millimeters of the ribs from the *Dahalokely tokana* holotype, UA 8678.

Rib	ML	CTL
**DR?2**	339.9	78.9
**DRcd**	†	58.8

**Abbreviations:** DR?2, dorsal rib ?2; Drcd, dorsal rib fragment figured in Figures 10C, 10D; ML, maximum length, from tuberculum to distal end of shaft; CTL, maximum distance between extreme ends of capitulum and tuberculum; †, element incomplete for measurement.

## Geological Context

### Age of the *Dahalokely tokana* holotype locality

Several studies have considered the age of the Upper Cretaceous section exposed in and around the area of Antsiranana using biostratigraphy [23]–[27]. Although the sediments that produced *Dahalokely* have not yet yielded fossils of biostratigraphic significance, these strata are bracketed by marine deposits containing biostratigraphically informative fossils.

The section containing the *Dahalokely* site is informally termed the “Ambolafotsy Formation,” and is divided into lower, middle (containing the type locality; Figure 1), and upper units [23]. The lower unit has produced several biostratigraphically informative foraminifera, including *Whiteinella aprica*, *W. baltica*, *Helvetoglobotruncana praehelvetica*, and *H. helvetica*, and the nannofossil *Quadrum gartneri* [23], [25], placing the sample within the *Q. gartneri* and *H. helvetica* zones [25]. Relevant biostratigraphic zones and ranges were sourced from Ogg and Lugowski [28], which draws upon data from other published resources (particularly references within [29]), recalibrated to the Geologic Time Scale 2012 [30]. *Helvetoglobotruncana helvetica* is the most informative of the foraminifera, defining a zone within the latter part of the early Turonian (∼93.52–92.99 Ma). The earliest occurrence of *Q. gartneri* is during the second half of the early Turonian (93.55 Ma), and it persisted into the late Coniacian (∼86.44 Ma). Thus, the co-occurrence of these two species suggests that the sediments of the lower unit were deposited during the latter part of the early Turonian (93.52–92.99 Ma). The upper unit contains the ammonite *Subprionocyclus neptuni* [23], [24], a taxon restricted to the late Turonian (90.86–89.77 Ma). Because no biostratigraphically informative microfossils are known from the middle unit yet, these terrestrial deposits may have overlapped temporally with some of the marine biozones. Even so, this biostratigraphic evidence restricts the age of the type locality for *Dahalokely* to the interval including the latter part of the early Turonian through the late Turonian (∼93.52–89.77 Ma).

### Depositional environment of the *Dahalokely tokana* holotype locality

The sediments of most of the middle unit of the Ambolafotsy Formation are interpreted as terrestrial, deposited during a marine regression [23]. Carbonized plant fragments are quite common in the middle unit, along with claystones, shales, and cross-bedded sandstones. Marine microfossils and macrofossils are generally absent [23], [25], although a deposit of ostreids several meters above the type locality for *Dahalokely* suggests that the area was deposited close to the shoreline.

### Systematic Paleontology

Theropoda Marsh 1881 [31] *sensu* Gauthier 1986 [32].

Abelisauroidea Bonaparte and Novas 1985 [33] *sensu* Bonaparte 1991 [34].

*Dahalokely* Farke and Sertich gen. nov.

urn:lsid:zoobank.org:act:8147803A-D4BE-4BA9-9701-D853E37DE411.

*Dahalokely tokana* Farke and Sertich sp. nov.

urn:lsid:zoobank.org:act:AFAE32BB-123A-45D4-B931-4FF2AABAF41C.

### Holotype

UA 9855, a partial axial column including cervical vertebra C?5, dorsal vertebrae D?1, D?2, and D?6–D?9, as well as a complete left dorsal rib (DR?2), capitula of two right dorsal ribs, proximal ends of two dorsal ribs, and other rib fragments (Figures 2, 3, 4, 5, 6, 7, 8, 9, and 10, S2, S3, S4, and S5). The elements were disarticulated but closely associated *in situ* within 1 m of each other.

**Figure 2:**
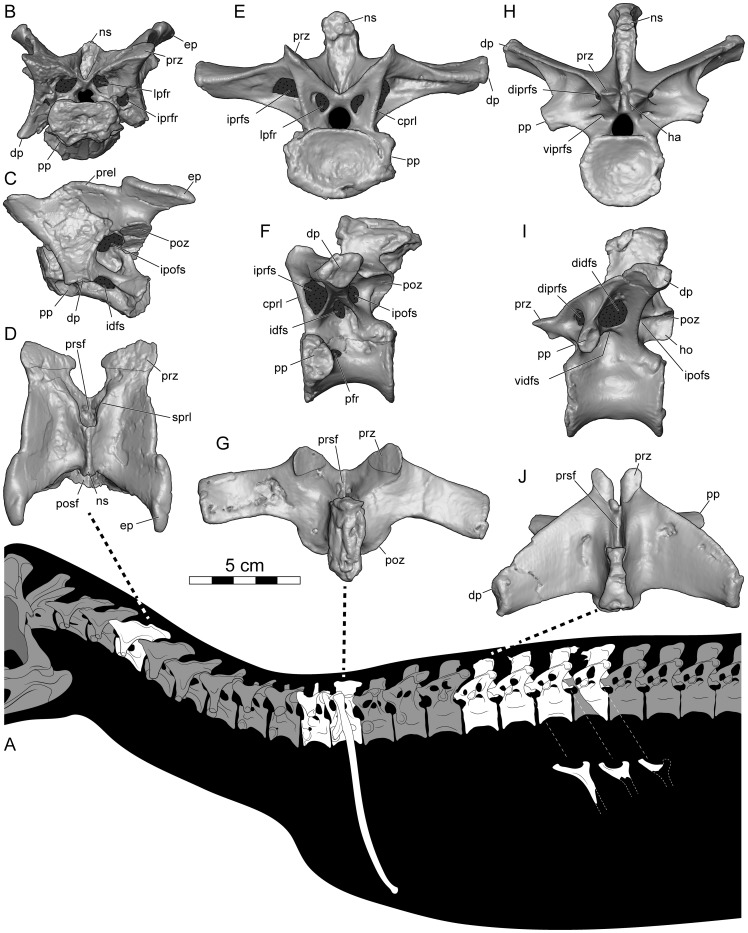
*Dahalokely tokana*, holotype (UA 9855). **A**, reconstructed silhouette with preserved elements indicated in white. Cervical vertebra (C?5) in **B**, cranial; **C**, left lateral; and **D**, dorsal views. Dorsal vertebra (D?2) in **E**, cranial; **F**, left lateral, and **G**, dorsal views. Dorsal vertebra (D?6) in **H**, cranial; **I**, left lateral; and **J**, dorsal views. Abbreviations: cprl, centroprezygapophyseal lamina; didfs, dorsal infradiapophyseal fossa; diprfs, dorsal infraprezygapophyseal fossa; dp, diapophysis; ep, epipophysis; ha, hypantrum; ho, hyposphene; idfs, infradiapophyseal fossa; ipofs, infrapostzygapophyseal fossa; iprfr, infraprezygapophyseal foramen; iprfs, infraprezygapophyseal fossa; lpfr, laminopeduncular foramen; ns, neural spine; pfr, pneumatic foramen; posf, postspinal fossa; poz, postzygapophysis; pp, parapophysis; prel, prezygoepipophyseal lamina; prsf, prespinal fossa; prz, prezygapophysis; sprl, spinoprezygapophyseal lamina; vidfs, ventral infradiapophyseal fossa; viprfs, ventral infraprezygapophyseal fossa.

**Figure 3:**
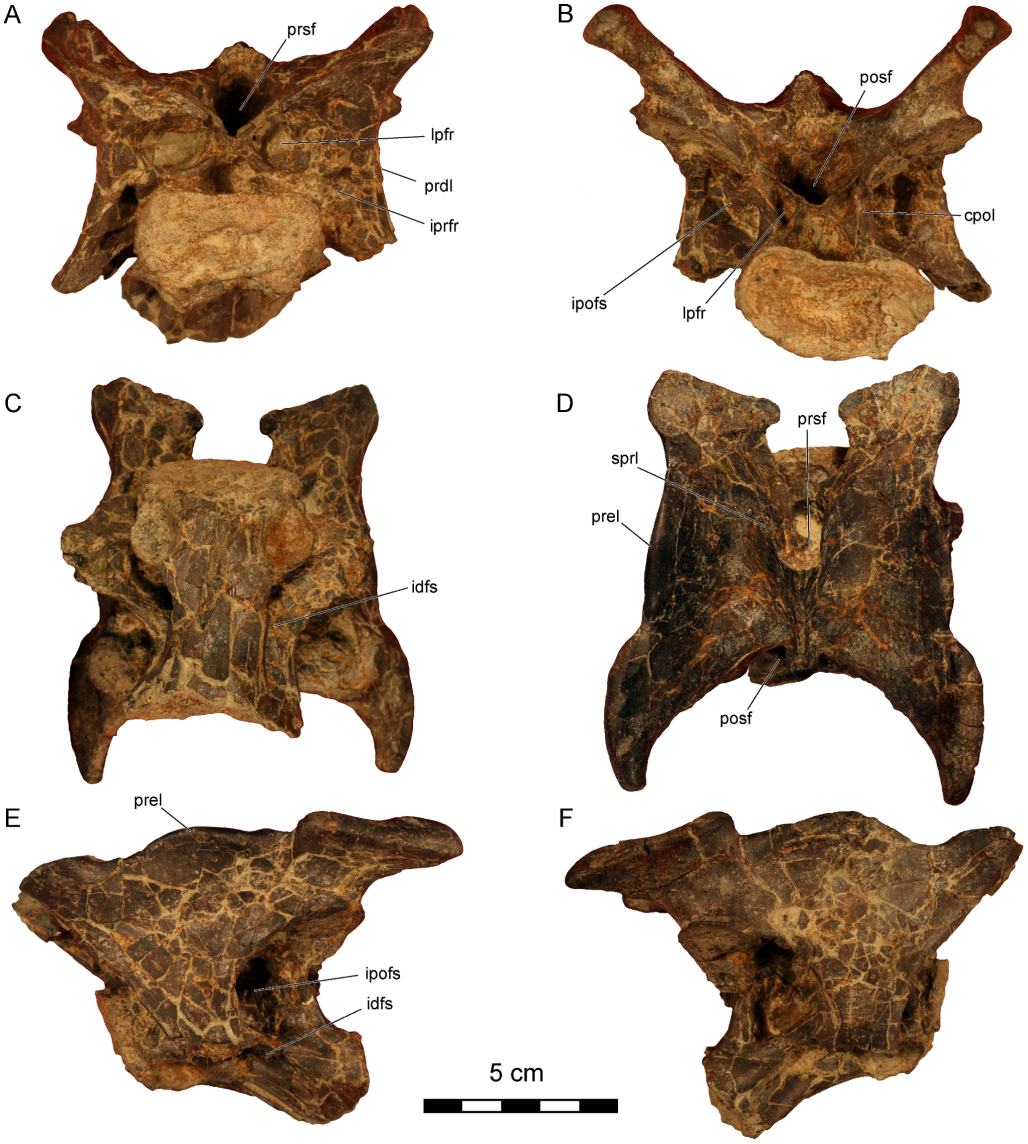
*Dahalokely tokana* holotype (UA 9855), ?fifth cervical (C?5) vertebra. Photographs in **A**, cranial; **B**, caudal; **C**, ventral; **D**, dorsal; **E**, left lateral; and **F**, right lateral views. Abbreviations: cpol, centropostzygapophyseal lamina; idfs, infradiapophyseal fossa; ipofs, infrapostzygapophyseal fossa; iprfr, infraprezygapophyseal foramen; lpfr, laminopeduncular foramen; posf, postspinal fossa; prdl, prezygodiapophyseal lamina; prel, prezygoepipophyseal lamina; prsf, prespinal fossa; sprl, spinoprezygapophyseal lamina.

**Figure 4:**
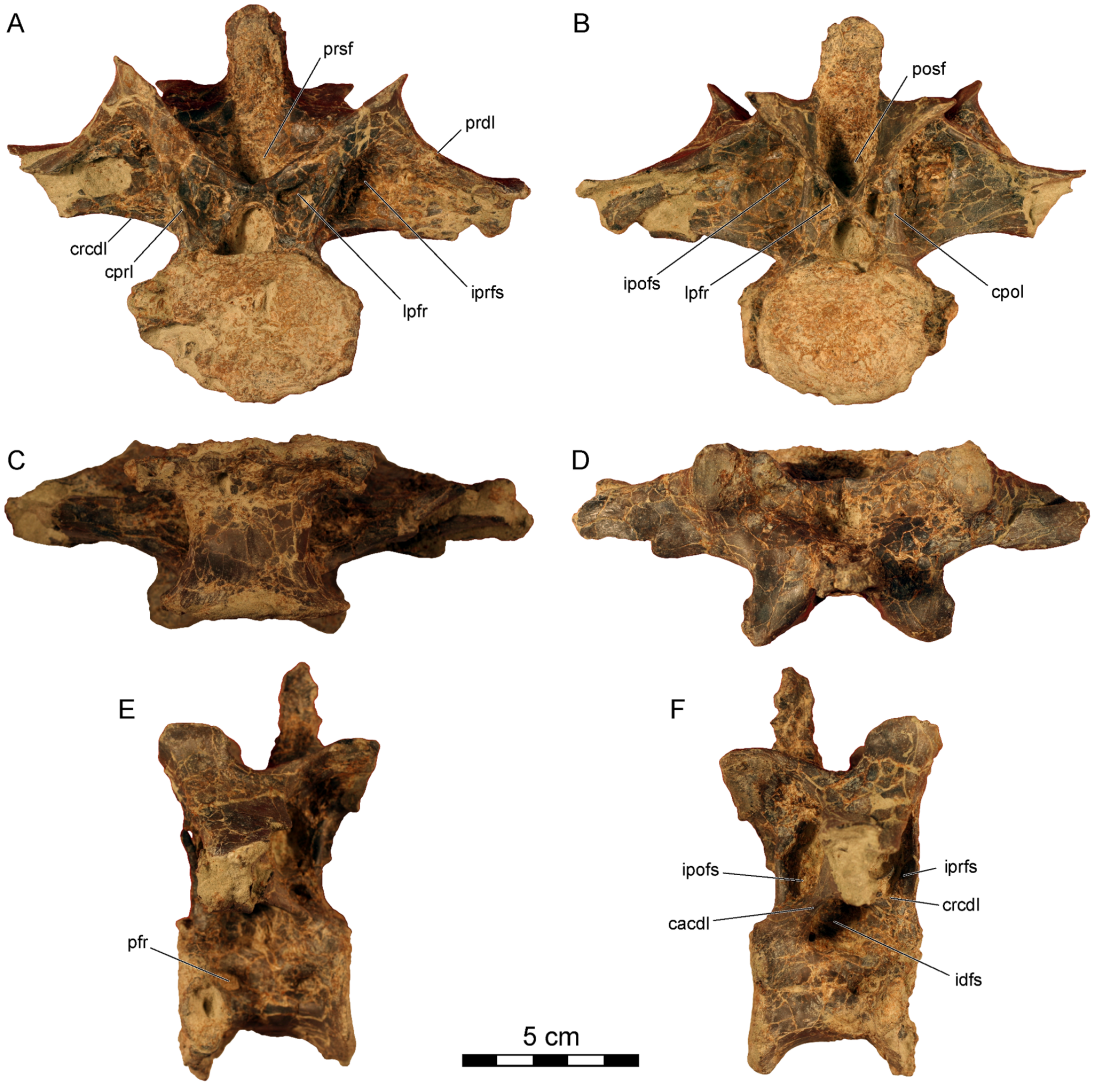
*Dahalokely tokana* holotype (UA 9855), ?first dorsal (D?1) vertebra. Photographs in **A**, cranial; **B**, caudal; **C**, ventral; **D**, dorsal; **E**, left lateral; and **F**, right lateral views. Abbreviations: cacdl, caudal centrodiapophyseal lamina; cpol, centropostzygapophyseal lamina; cprl, centroprezygapophyseal lamina; crcdl, cranial centrodiapophyseal lamina; idfs, infradiapophyseal fossa; ipofs, infrapostzygapophyseal fossa; iprfs, infraprezygapophyseal fossa; lpfr, laminopeduncular foramen; pfr, pneumatic foramen; posf, postspinal fossa; prdl, prezygodiapophyseal lamina; prsf, prespinal fossa.

**Figure 5:**
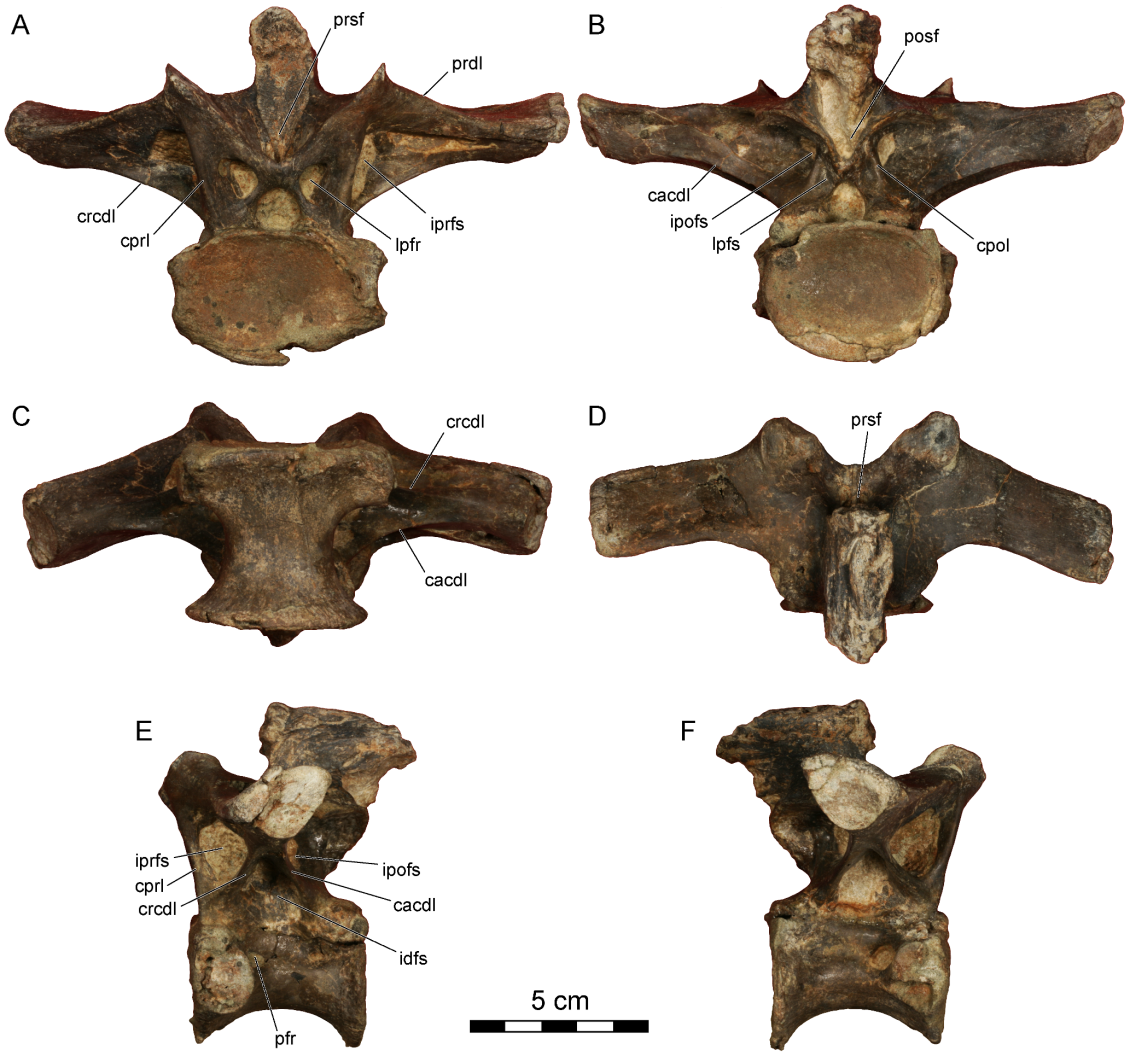
*Dahalokely tokana* holotype (UA 9855), ?second dorsal (D?2) vertebra. Photographs in **A**, cranial; **B**, caudal; **C**, ventral; **D**, dorsal; **E**, left lateral; and **F**, right lateral views. Abbreviations: cacdl, caudal centrodiapophyseal lamina; cpol, centropostzygapophyseal lamina; cprl, centroprezygapophyseal lamina; crcdl, cranial centrodiapophyseal lamina; idfs, infradiapophyseal fossa; ipofs, infrapostzygapophyseal fossa; iprfs, infraprezygapophyseal fossa; lpfr, laminopeduncular foramen; lpfs, laminopeduncular fossa; pfr, pneumatic foramen; posf, postspinal fossa; prdl, prezygodiapophyseal lamina; prsf, prespinal fossa.

**Figure 6:**
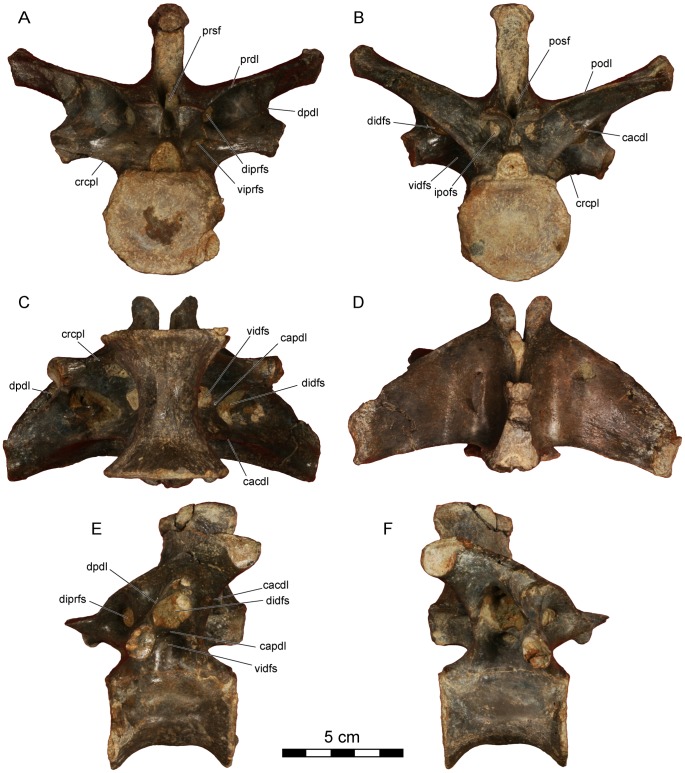
*Dahalokely tokana* holotype (UA 9855), ?sixth dorsal (D?6) vertebra. Photographs in **A**, cranial; **B**, caudal; **C**, ventral; **D**, dorsal; **E**, left lateral; and **F**, right lateral views. Abbreviations: cacdl, caudal centrodiapophyseal lamina; capdl, caudal paradiapophyseal lamina; crcpl, cranial centroparapophyseal lamina; didfs, dorsal infradiapophyseal fossa; diprfs, dorsal infraprezygapophyseal fossa; dpdl, dorsal paradiapophyseal lamina; ipofs, infrapostzygapophyseal fossa; podl, postzygadiapophyseal lamina; posf, postspinal fossa; prdl, prezygodiapophyseal lamina; prsf, prespinal fossa; vidfs, ventral infradiapophyseal fossa; viprfs, ventral infraprezygapophysesal fossa.

**Figure 7:**
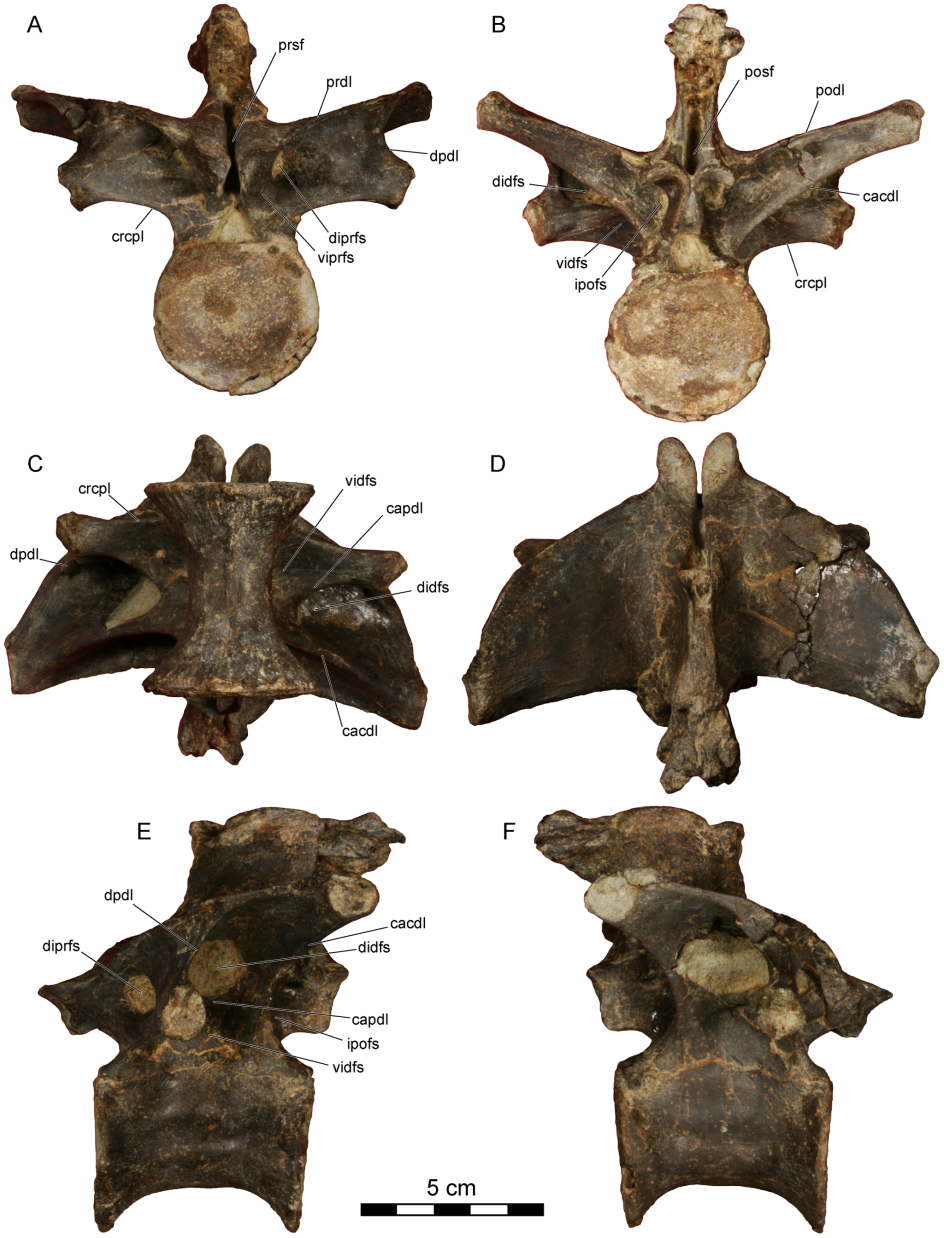
*Dahalokely tokana* holotype (UA 9855), ?seventh dorsal (D?7) vertebra. Photographs in **A**, cranial; **B**, caudal; **C**, ventral; **D**, dorsal; **E**, left lateral; and **F**, right lateral views. Abbreviations: cacdl, caudal centrodiapophyseal lamina; capdl, caudal paradiapophyseal lamina; crcpl, cranial centroparapophyseal lamina; didfs, dorsal infradiapophyseal fossa; diprfs, dorsal infraprezygapophyseal fossa; dpdl, dorsal paradiapophyseal lamina; ipofs, infrapostzygapophyseal fossa; podl, postzygadiapophyseal lamina; posf, postspinal fossa; prdl, prezygodiapophyseal lamina; prsf, prespinal fossa; vidfs, ventral infradiapophyseal fossa; viprfs, ventral infraprezygapophysesal fossa.

**Figure 8:**
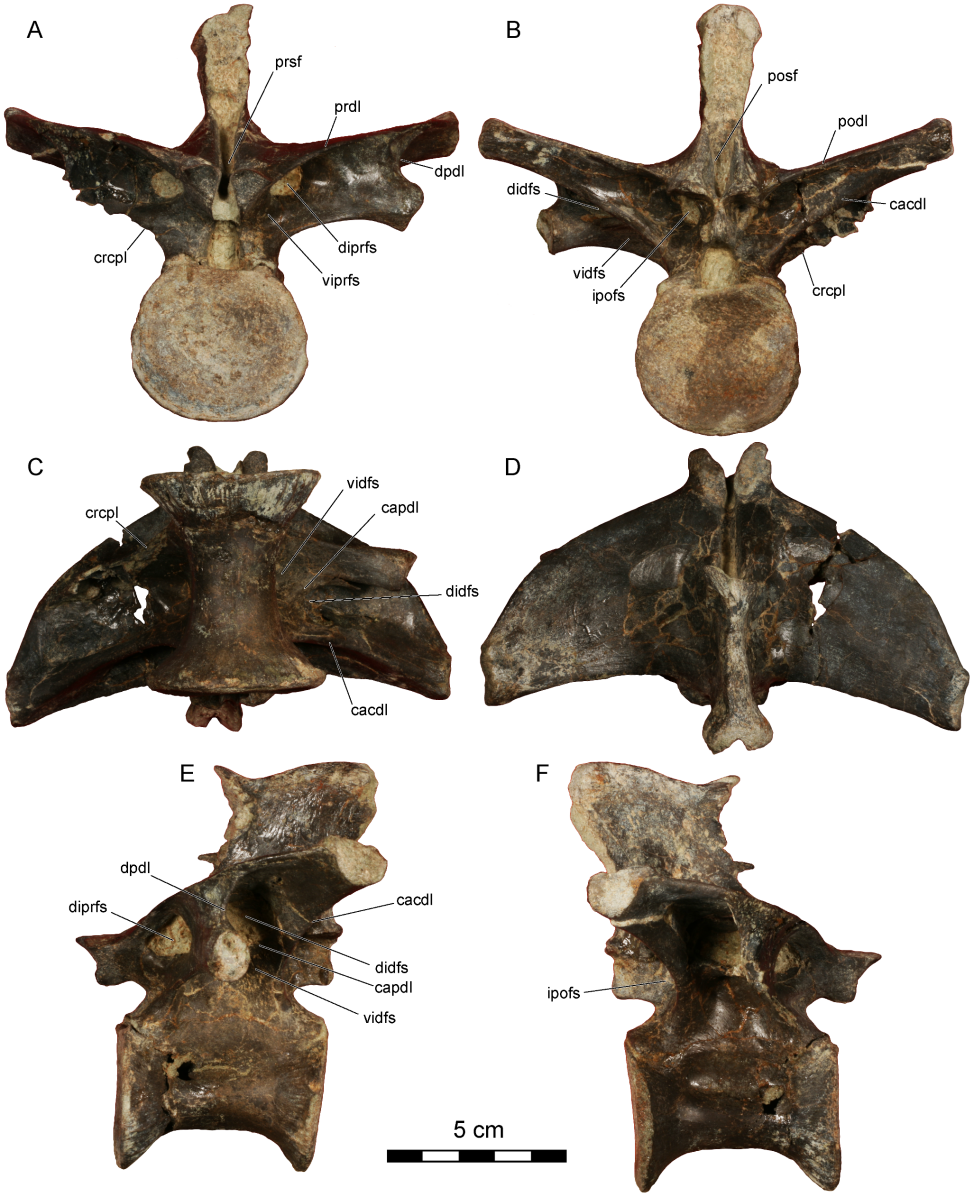
*Dahalokely tokana* holotype (UA 9855), ?eighth dorsal (D?8) vertebra. Photographs in **A**, cranial; **B**, caudal; **C**, ventral; **D**, dorsal; **E**, left lateral; and **F**, right lateral views. Abbreviations: cacdl, caudal centrodiapophyseal lamina; capdl, caudal paradiapophyseal lamina; crcpl, cranial centroparapophyseal lamina; didfs, dorsal infradiapophyseal fossa; diprfs, dorsal infraprezygapophyseal fossa; dpdl, dorsal paradiapophyseal lamina; ipofs, infrapostzygapophyseal fossa; podl, postzygadiapophyseal lamina; posf, postspinal fossa; prdl, prezygodiapophyseal lamina; prsf, prespinal fossa; vidfs, ventral infradiapophyseal fossa; viprfs, ventral infraprezygapophysesal fossa.

**Figure 9:**
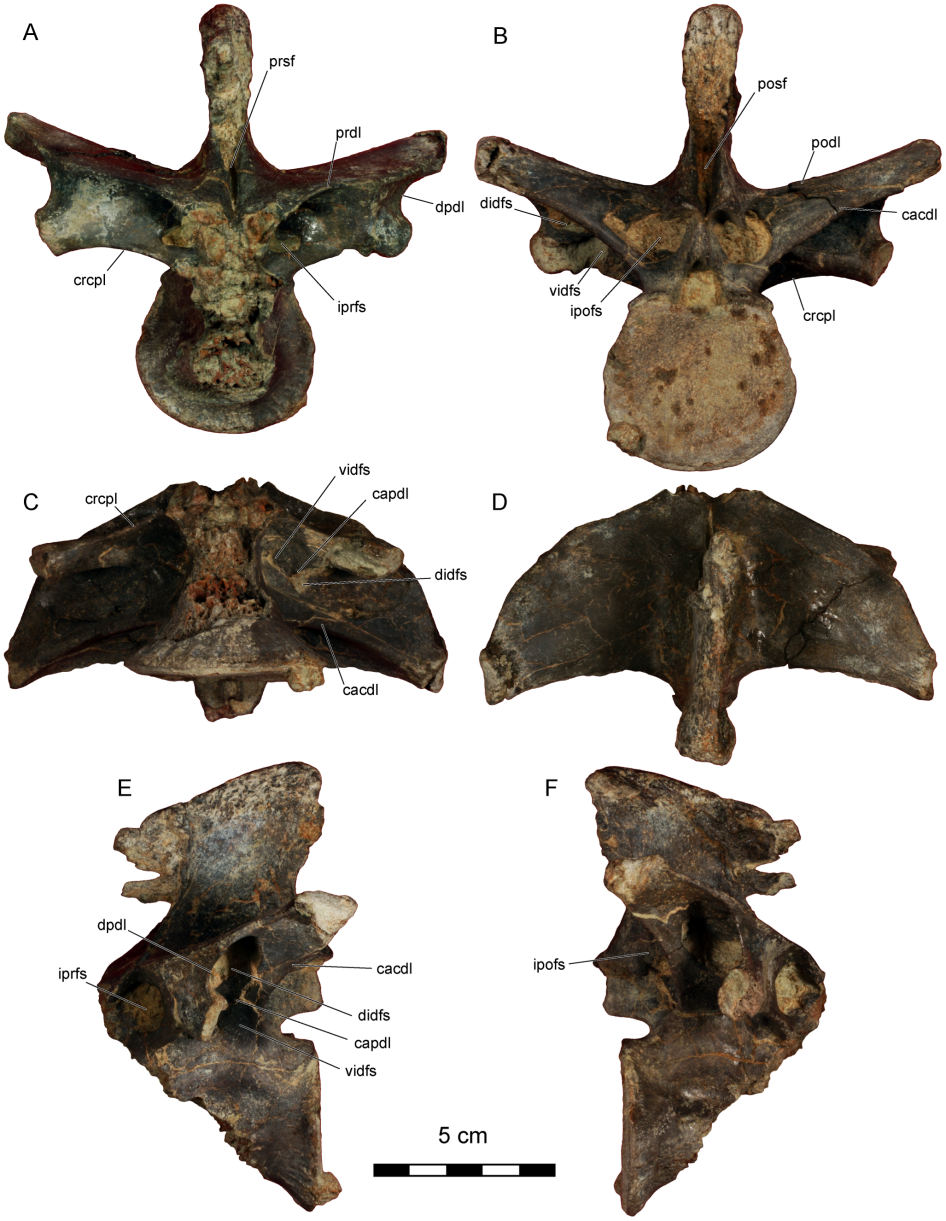
*Dahalokely tokana* holotype (UA 9855), ?ninth dorsal (D?9) vertebra. Photographs in **A**, cranial; **B**, caudal; **C**, ventral; **D**, dorsal; **E**, left lateral; and **F**, right lateral views. Abbreviations: cacdl, caudal centrodiapophyseal lamina; capdl, caudal paradiapophyseal lamina; crcpl, cranial centroparapophyseal lamina; didfs, dorsal infradiapophyseal fossa; dpdl, dorsal paradiapophyseal lamina; ipofs, infrapostzygapophyseal fossa; iprfs, infraprezygapophysesal fossa; podl, postzygadiapophyseal lamina; posf, postspinal fossa; prdl, prezygodiapophyseal lamina; prsf, prespinal fossa; vidfs, ventral infradiapophyseal fossa.

**Figure 10:**
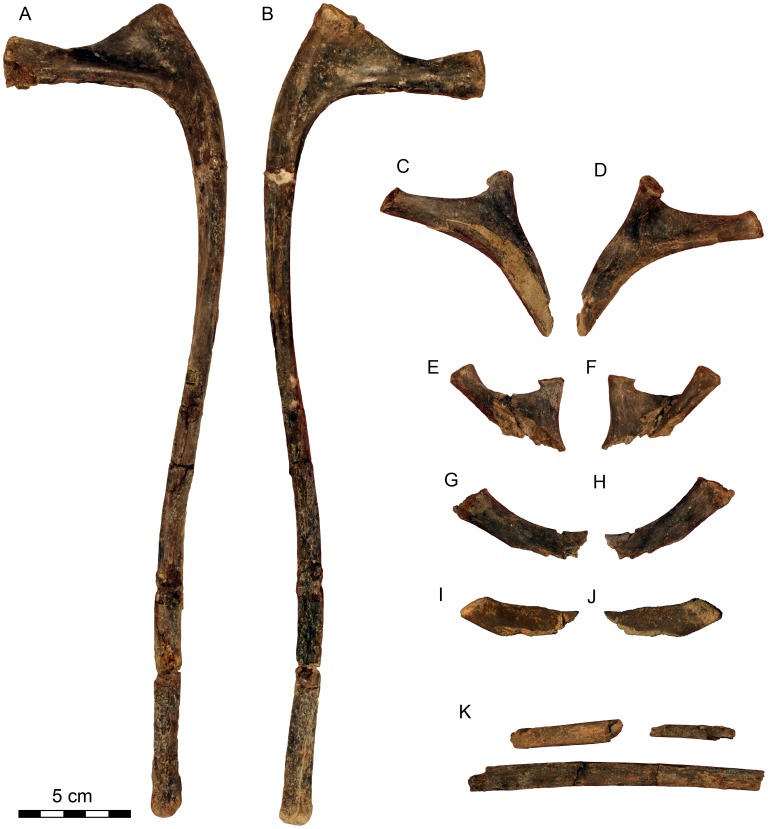
*Dahalokely tokana* holotype (UA 9855), dorsal ribs. ? Second dorsal rib (DR?2) of *Dahalokely tokana* in **A**, craniolateral; and **B**, caudomedial views. Proximal ends of dorsal ribs in craniolateral (**C**, **E**, **G**, **I**) and caudomedial (**D**, **F**, **H**, **J**) views; **K**, other rib fragments associated with UA 9855.

### Etymology

The generic name, from the Malagasy *dahalo* (bandit) and *kely* (small), references the small size of the animal relative to many abelisauroids. The species epithet, *tokana* (Malagasy, lonely), references the organism’s isolation on the landmass of Indo-Madagascar. The suggested, generalized pronunciation based on the Malagasy language is “dah-HAH-loo-KAY-lee too-KAH-nah.”

### Locality and horizon

Near Ampandriambengy, within the Diego (Ambilobe) Basin of northernmost Madagascar, from the middle, terrestrial unit of the “Ambolafotsy Formation,” Turonian in age ([23]; see Geology, below; Figure 1). Precise locality data, available to qualified researchers, are archived at University of Antananarivo and Stony Brook University.

### Diagnosis

An abelisauroid theropod characterized by the following autapomorphies: mid-cervical vertebrae have prezygoepipophyseal lamina with prominent convexity at midpoint nearly equal in length to centrum and prominent notches at either end separating convexity from epipophyses; prezygapophyses and centroprezygapophyseal lamina nearly vertical and linear in lateral view in D1 and D2, with cranial margin of prezygapophyses and cranial face of centrum nearly co-planar; postzygapophyses on D2 strongly concave; infraprezygapophyseal fossa divided through D6.

### Differential diagnosis

In addition to the autapomorphies listed in the diagnosis, *Dahalokely tokana* is differentiated from other abelisauroids by a unique combination of apomorphies. Because many vertebral positions are unknown both for *D. tokana* and other abelisauroids, the number of apomorphies is almost certainly broader than indicated here. Comparable elements are not yet known for the abelisaurids *Abelisaurus comahuensis*, *Indosaurus matleyi*, *Indosuchus raptorius*, *Kryptops palaios*, *Pycnonemosaurus nevesi*, *Quilmesaurus curriei*, and *Rugops primus*, so these taxa cannot be morphologically differentiated from *D. tokana* (or indeed, many other recognized abelisaurid taxa) at present.

*Dahalokely tokana* has smaller vertebrae (and thus presumably smaller body size) than all known abelisaurids.

Relative to *Aucasaurus garridoi*, *Dahalokely tokana* has D?1 and D?2 vertebrae with more elongate centra and relatively shorter, more caudally-placed neural spines.

Relative to *Carnotaurus sastrei*, *Dahalokely tokana* has mid-cervical vertebrae with rectangular rather than triangular transverse processes, no cranial extension of the epipophyses, a more strongly dorsoventrally compressed centrum, more cranially-placed prezygapophyses and more caudally-placed postzygapophyses, the line defined by the tips of the epipophyses and prezygapophyses is more closely parallel to the ventral edge of the centrum, the neural spine is shorter and triangular rather than rectangular in cross-section, the prespinal fossa is more deeply excavated with a more elongated bounding notch, and the cranial laminopeduncular foramina are relatively smaller. The D?1 and D?2 vertebrae of *D. tokana* are more strongly dorsoventrally compressed, with weakly amphicoelus (rather than procoelus) centra and more horizontally directed transverse processes. In the mid-dorsal series, the centra are relatively more elongate, the transverse processes are triangular in dorsal profile rather than rectangular, and the transverse processes are more horizontally oriented, resulting in a lower parapophysis relative to the neural canal.

Relative to *Ekrixinatosaurus novasi*, *Dahalokely tokana* has more strongly dorsoventrally compressed and elongate centra in mid-cervical vertebrae, and lacks a prominent caudal centrozygapophyseal fossa.

Relative to *Ilokelesia aguadagrandensis*, *Dahalokely tokana* has relatively more elongate and more dorsoventrally-compressed centra on mid-cervical vertebrae.

Relative to *Majungasaurus crenatissimus*, the mid-cervical vertebrae of *Dahalokely tokana* have centra that are more strongly dorsoventrally compressed with convergent cranial and caudal articular surfaces in lateral view. The notch between the prezygapophyses is deeper, the postzygapophyses are more cranially placed relative to the centrum, and the caudal centrodiapophyseal lamina and infrapostzygapophyseal fossa are more extensive. Within the cranial dorsal vertebrae of *Dahalokely*, the centra are wider and more dorsoventrally compressed, the prezygapophyseal facets on D?1 are oriented at 45 degrees from vertical rather than being horizontal, and the prezygapophyseal facets on both D?1 and D?2 are triangular (rather than oval) and more smoothly confluent with the transverse processes and laminoprezygapophyseal lamina in dorsal view. Within mid- to caudal dorsal vertebrae, the hyposphene and hypantra are strongly divergent ventrally rather than parallel, the transverse processes of D?5 and D?6 are less strongly back-swept, and the prezygapophyses are cranio-caudally elongated rather than mediolaterally elongated. For the second dorsal rib (DR?2) in *Dahalokely tokana*, the tuberculum is more indistinct relative to the capitulotubercular web than for the equivalent rib in *M. crenatissimus*.

Relative to *Rahiolisaurus gujaratensis*, the centra of the mid-cervical vertebrae in *Dahalokely tokana* are more strongly dorsoventrally compressed.

As compared to *Rajasaurus narmadensis*, the postzygapophyses of cranial dorsal vertebrae are mediolaterally rather than craniocaudally elongated, and a cranial laminopeduncular fossa occurs in *Dahalokely tokana*.

Relative to *Skorpiovenator bustingorryi*, the transverse process of the cervical vertebrae is rectangular rather than triangular in *Dahalokely tokana*.

Relative to *Xenotarsosaurus bonapartei*, the centra of the cranial dorsal vertebrae are more dorsoventrally compressed and craniocaudally elongated in *Dahalokely tokana*.

Among noasaurids, only *Masiakasaurus knopfleri* is known from adequate, confidently assigned vertebral material that overlaps with the holotype of *Dahalokely tokana*. Relative to *M. knopfleri*, *Dahalokely tokana* is larger and has more caudally-placed neural spines on all vertebrae. The mid-cervical vertebrae of *Dahalokely tokana* have mediolaterally rather than craniocaudally elongated prezygapophyses, square rather than rectangular neural arches in dorsal view, and much more prominent epipophyses (extending well caudal and lateral to the postzygapophyses). The D?1 vertebra has a a relatively shorter centrum, prominent laminopeduncular fossae (rather than being absent), dual pneumatic foramina on the centrum, and lack of a hyposphene. In mid- to caudal dorsal vertebrae, the neural spines of *D. tokana* are more uniform in mediolaterally width along their craniocaudal extent, the centra are more prominently spool-shaped (with relatively broader cranial and caudal faces as compared to the centrum length), and the hyposphene/hypantrum articulations are more equal in size to the zygapophyses (rather than being much smaller than the zygapophyses in *M. knopfleri*). Cranial dorsal ribs are not pneumatized in *D. tokana*, but are pneumatized in *M. knopfleri*.

### Description

The vertebrae of UA 9855 were identified to position within the vertebral column by comparison with a nearly complete presacral vertebral series preserved for *Majungasaurus* [22]. Neurocentral sutures are well coossified on the cervical vertebra and firmly fused but still visible on the caudal dorsal vertebrae. This suggests that the individual had achieved nearly full adult size but had not yet reached full skeletal maturity [22]. In the following description, comparisons focus on *Carnotaurus*, *Majungasaurus*, and *Masiakasaurus*, abelisauroids for which the best-preserved and most thoroughly-described vertebral series are known. Additional comparisons with other abelisauroids are included as appropriate; the differential diagnoses (above) also contain a species-by-species comparison of relevant characters.

The centrum on the cervical vertebra (C?5) is 65 mm long and strongly dorsoventrally compressed (width:height = 2), with strongly concave caudal and relatively flat cranial articular surfaces (Figures 2B–D, 3, S1). In lateral view, the articular surfaces are not parallel as in *Majungasaurus*, but similar to other abelisauroids (Figures 2B, 3E, 3F; e.g., *Masiakasaurus* or *Carnotaurus* [18], [35]). Two subdued parasagittal ridges extend caudally from the parapophyses on the ventral surface of the vertebra, similar to the condition in other abelisauroids (e.g., *Carnotaurus*, *Majungasaurus*, and *Masiakasaurus*). A prominent fossa, associated with the infrapostzygapophyseal foramen, occupies the dorso-lateral surface of the centrum (Figure 2C). The caudal centrodiapophyseal lamina is marked, intersecting with the centrum at its caudal third, unlike *Majungasaurus* or *Carnotaurus*, but similar to *Ilokelesia* and *Masiakasaurus* [22], [35], [36]. A prominent pleurocoel, the infradiapophyseal fossa, invaginates the centrum immediately ventral to the aforementioned lamina (Figure 2C). Two foramina (presumably pneumatic in origin) occur within this fossa on both sides; the cranial one is largest. Overall, the fossa is similar in morphology to that in other abelisauroids.

In dorsal view, the overall profile of the cervical vertebra is relatively square (Figures 2D, 3D), similar to other abelisaurids but contrasting sharply with the elongate cervical vertebrae of noasaurids. The tips of the prominent epipophyses are elongated relative to the condition in *Majungasaurus* or *Masiakasaurus*, but similar to that of *Carnotaurus* [21], [37]. This may be due at least in part to individual or ontogenetic variation. The cranial edge of each epipophysis has a prominent bulge, but it does not form a discrete cranial process as in many other taxa. The dorsal margin of the prezygoepiphoyseal lamina has a prominent convexity at its midpoint, unlike the straight, concave, or only slightly convex condition seen in other abelisauroids. Prominent notches separate the convexity from the epipophysis and prezygapophysis; as measured between the notches, the convexity is approximately the same length as the centrum. This convexity completely obscures the dorsoventrally low and cranio-caudally elongated neural spine in lateral view. A cervical vertebra of the *Ilokelesia* holotype (MCF-PVPH-35; Museo Municipal Carmen Funes, Plaza Huincul, Argentina) shows a similar morphology, but bone texture in the area (gnarled and irregular with a sharply angled dorsal margin, rather than smooth) suggests it may be pathological. Moreover, other known cervicals for the *Ilokelesia* holotype, presumably from adjacent positions, are more similar to those of *Majungasaurus* [22], [36]. Additionally, the neural spine of UA 9855 is triangular in cross-section (Figures 2B, 3A, 3B), similar to the condition in *Masiakasaurus* but differing from the squared profile of *Carnotaurus* and the elongated and rounded profile of *Majungasaurus*. The lateral margins of the caudal half of the prespinal fossa are parallel in UA 9855 (Figure 2D), rather than convergent as seen in *Majungasaurus*, *Masiakasaurus*, and *Carnotaurus*, and the postspinal fossa is undivided, unlike *Majungasaurus*. In lateral view, the transverse processes taper only gently ventrad, and the cranial and caudal borders are nearly parallel at the distal half of the process (Figures 2C, 3E, 3F), similar to the condition in *Majungasaurus* and differing from the sharply tapering, triangular shape in South American abelisaurids (e.g., *Skorpiovenator* and *Carnotaurus*). Prominent laminopeduncular foramina occur both cranially and caudally (Figures 2B, 3A, 3B), both in the cervical vertebra as well as D?1 and D?2. In *Majungasaurus*, cranial and caudal foramina occur through the level of C9, and cranial foramina alone occur through the level of D4. Cranial and caudal foramina or fossae occur at least to the level of D2 in *Carnotaurus*, but only to C4 in *Masiakasaurus*. The prezygodiapophyseal lamina of C?5 in *Dahalokely* is much straighter in lateral view than in *Majungasaurus* or *Carnotaurus*. The zygapophyses are mediolaterally expanded; the prezygapophyses taper to a point medially (Figure 2D), whereas the taper is less pronounced on the postzygapophyses. The parapophyses are roughly circular and directed ventro-laterally.

All dorsal centra are amphicoelus, with D?1 and D?2 (centrum lengths of 44 and 49 mm, respectively) more strongly so than D?6–D?9 (centrum lengths of 52 mm for D6 and 55 mm each for D?7 and D?8). The first two vertebrae (D?1 and D?2) are identified as dorsals because the parapophysis is enlarged and shifted dorsally relative to condition in the cervical vertebra, the transverse processes are elevated and subhorizontal, and epipophyses are absent [22]. The parapophyses are placed at approximately mid-centrum height, below the level of the neuro-central suture. Centra are both wider and longer than they are tall (Figures 2E–G, 4, 5, S2, S3; unlike *Majungasaurus* and *Carnotaurus* but similar to *Rajasaurus* and *Masiakasaurus* [13], [18], [21], [22]) and lack a ventral ridge. The erect, mediolaterally expanded neural spine is located over the caudal half of the vertebra (Figures 2F, 4E, 4F, 5E, 5F). In D?2 (Figures 5, S3), the distal half of the neural spine is associated with hyperossified supraspinous ligaments that are most prominent on the caudal surface. Similar to other abelisauroids for which the condition is known (e.g., *Carnotaurus*, *Majungasaurus*), the spine on D?1 is craniocaudally shorter than the spine on D?2. A large, cranially-placed pneumatic foramen occurs just caudal to the parapophysis on D?2 (Figures 2F, 5E, 5F, S3), whereas both cranially and caudally-placed foramina occur bilaterally in D?1 (Figures 4E, 4F, S2). The transverse processes are directed laterally, as in *Majungasaurus*, *Allosaurus*, and many other theropods. Both pre- and postzygapophyseal facets are oriented at 45° relative to the vertical in D?1 (Figure 4A,B), whereas only the prezygapophyseal facets are so oriented in D?2 (Figures 2E, 5A). Here, the postzygapophyseal facets are oriented in a near-horizontal fashion (Figure 5B). In *Majungasaurus* and *Carnotaurus*, both the pre- and postzygapophyses are oriented at 45° in D?2. Unusually, the dorsal extents of the prezygapophyses are markedly higher than the dorsal extents of the postzygopophyses when the ventral margin of the centrum is placed horizontally in UA 9855. In other abelisauroids for which the condition is known, the zygapophyses are at roughly the same dorso-ventral level. The cranial borders of the prezygapophyses are smoothly continuous with the lamina in UA 9855 (Figures 2F, 4E, 4F, 5E, 5F), as in *Carnotaurus*, rather than having a sharp cranial projection as seen in *Majungasaurus*. D?2 has an incipient hyposphene (Figure 5B), also as in *Majungasaurus*; the feature occurs on D1 in *Masiakasaurus* [18]. In cranial view, the centroprezygapophyseal lamina intersects the prezygapophysis at its midpoint like in *Carnotaurus* and *Masiaksaurus* (Figure 2F), rather than the medially-placed intersection of the lamina in *Majungasaurus* [22]. In lateral view, the centroprezygapophyseal laminae of D?1 and D?2 are nearly vertical in UA 9855, contrasting with the canted orientation of other abelisauroids (e.g., *Carnotaurus*, *Majungasaurus*, and *Masiakasaurus*). Consequently, the cranial edges of the prezygapophysis and centrum are approximately co-planar. A posterior cervical (C?10) of *Ekrixinatosaurus* (MUCPv-294 [Museo Universidad Nacional del Comahue, Paleovertebrados, Neuquen, Argentina]) displays somewhat similar morphology, but the cranioventral edge of the lamina is slightly set back from the centrum, unlike the condition in *Dahalokely*. Similar to the condition in *Majungasaurus*, infraprezygapophyseal, infradiapophyseal, and infrapostzygapophyseal fossae are prominent in UA 9855 (Figure 2E, 2F). The transverse process in D?2 is smoothly continuous with the prezygapophysis in dorsal view, rather than a stepped as in *Majungasaurus*.

The centra of vertebrae D?6–D?9 (Figures 2H–J, 6, 7, 8, 9, S4, S5) are apneumatic, subcircular in articular view, strongly constricted at their midpoints, roughly 20% longer than wide, and exhibit a low midline keel on the ventral surfaces. The neural arches are extremely pneumatic, with the infradiapophyseal fossae of all vertebrae split into dorsal and ventral portions by a caudal paradiapophyseal lamina (Figures 2I, 6E, 6F, 7E, 7F, 8E, 8F, 9E, 9F), as is typical for abelisauroids. In D?6 (Figures 2H–J, 6, S4), the ventral portion is further subdivided into an cranial and caudal fossa on the right side only. The infrapostzygapophyseal fossa is quite prominent and more deeply excavated than that in *Majungasaurus* [22]. In D?6, a stout lamina divides the infraprezygapophyseal fossa into a larger dorsal portion and a smaller ventral portion. No division exists in D?7–D?9, although a faint ventral depression is visible in D?7 and D?8 (Figures 8E, 8F, 9E, 9F). The prezygapophyses of D?6–D?9 are much longer than wide (Figures 2J, 6D, 7D, 8D, 9D); this is similar to the condition (where known) in *Masiakasaurus*. These processes are wider than long in equivalent vertebrae of *Majungasaurus* and variable in *Carnotaurus*. By contrast, the articular surfaces of the postzygapophyses are slightly wider than long. Prominent hyposphenes and hypantra occur on D?6–D?9; unlike *Majungasaurus* or *Masiakasaurus* but similar to *Ilokelesia*, these structures are strongly divergent ventrally [22], [36].

In dorsal view, the transverse processes of D?6–D?9 are less swept-back than in *Majungasaurus*, particularly in D?6 (Figures 2J, 6D, 7D, 8D, 9D). The general shape of the processes is similar to the condition in *Majungasaurus* and *Masiakasaurus* in being relatively triangular, contrasting with the more quadrangular profile with a rounded craniolateral edge seen for equivalent positions in *Carnotaurus*. The cranial borders of the transverse processes in D?7–D?9 are strongly convex (similar to *Majungasaurus* and other abelisauroids), whereas the caudal borders are weakly concave in comparison to *Majungasaurus*, being more similar to the condition in *Masiakasaurus* or *Carnotaurus*. In D?6, the cranial and caudal borders are almost straight, unlike the morphology in *Masiakasaurus*. In all of these more caudal dorsals, the parapophyses are nearly entirely hidden by the transverse processes in dorsal view (similar to *Majungasaurus* but unlike *Carnotaurus*). The diapophyses are largest on D?6, steadily decreasing in size caudally. In cranial view, the transverse processes are oriented at 75–80° to the neural spine, similar to *Majungasaurus* but contrasting with the smaller angulation seen in *Carnotaurus* [21], [22]. As in other abelisauroids, the parapophyses are ventrally displaced from the transverse process. The parapophyses are immediately caudal to the cranial border of the centrum in D?6 (Figure 2I), and are located more caudally in each successive vertebra (Figures 6E, 6F, 7E, 7F, 8E, 8F, 9E, 9F). In D?9, the process is just cranial to the midpoint of the centrum. The cranial centroparapophyseal lamina and the caudal and dorsal paradiapophyseal laminae radiate from each parapophysis. The lateral edge of the dorsal paradiapophyseal laminae is caudally inclined in lateral view (except for D?7), and is strongly concave in cranial view.

The neural spines of D?6–D?9 are located over the caudal half of the centrum, with the cranial portion of the base gently grading into the prezygapophyses (Figures 6E, 6F, 7E, 7F, 8E, 8F, 9E, 9F). Distinct pre- and postspinal fossae are present, as well as rugose surfaces for attachment of the interspinous ligaments (most prominent on the dorsal half of the fossae). A portion of the ligaments are ossified on the cranial surface of the neural spine in D?9 (Figure 9E, 9F) and the caudal surface of the neural spine in D?8 (Figure 8E, 8F). In dorsal view, the cranial and caudal ends of the neural spines are expanded, with the caudal end approximately twice the width of the cranial end (Figures 2J, 6D, 7D, 8D, 9D). In this aspect, as well as the cranio-caudal expansion of the neural spines, vertebrae of UA 9855 are more similar to *Majungasaurus* than to other abelisaurids [22].

Dorsal ribs (DR) were identified to position based on articular congruence with the preserved vertebrae (when possible) and by comparison with *Majungasaurus* (Figures 2A, 10). All ribs are apneumatic, as in abelisaurids but unlike *Masiakasaurus*, in which the cranial dorsal ribs are at least partially pneumatized [18], [22]. The left DR?2 is complete and uncrushed (Figures 10A, 10B; Table 2). In craniolateral view (Figure 10A), the capitulum is robust and expanded slightly at its articular end, with the tuberculum indistinct relative to the capitulotubercular web (unlike *Majungasaurus*, but similar to *Carnotaurus* and many tetanuran theropods [22]). Whereas the rib shaft is straight in cranial view, the distal half is caudally directed in lateral view. In dorsal view, the rib shaft has a counterclockwise twist. Both cranial and caudal surfaces exhibit prominent intercostal ridges, with the ridge more pronounced on the cranial side. The capitula and tubercula on more caudal ribs (Figure 10C–J) are generally smaller than in DR?2, and the tuberculum is more prominently separated from the capitulotubercular web than in the former (similar to *Majungasaurus*, but unlike *Carnotaurus* and many tetanuran theropods [22]).

### Phylogenetic analysis

In order to assess the phylogenetic relationships of *Dahalokely tokana*, we modified previously published character matrices spanning Ceratosauria [16], [17]; a total of 30 taxa and 192 characters were analyzed (see Text S1 for details; data available in Dataset S1). Due to differences in character interpretation or new data, a small number of character codings were adjusted in the matrix relative to the original scorings from Carrano and Sampson [16] and Pol and Rauhut [17]. For character 6 (proportions/presence of the rostral maxillary ramus), *Ekrixinatosaurus novasi*, *Rugops primus*, *Masiakasaurus knopfleri*, and *Noasaurus leali* were scored as 2 (tall and blunt rostral ramus) from the original coding of 0 (rostral ramus absent). For character 25 (rostro-caudal length of postorbital relative to height), the scoring for *Ilokelesia aguadagrandensis* was changed from 0 (markedly less) to 1 (equal to or greater).

*Berberosaurus liassicus, Camarillasaurus cirugedae, Eoabelisaurus mefi, Rahiolisaurus gujaratensis,* and *Skorpiovenator bustingorryi* were coded from photographs and published descriptions [17], [38]–[41], whereas *Kryptops palaios* and *Dahalokely tokana* were coded from first-hand examination of specimens supplemented by photographs. Previously unscored characters for other taxa also were coded from photographs and published descriptions. *Austrocheirus isasii* was not included because it is highly incomplete for the characters considered here and because recent work suggests it is referable to Theropoda indeterminate rather than confidently being constrained as a ceratosaur [42].

*Berberosaurus liassicus* was hypothesized as a subadult individual [41], and thus ontogeny-dependent characters (particularly relating to the fusion between astragalus, tibia, and fibula) were coded as unknown.

*Rahiolisaurus gujaratensis* [40] is the fifth large-bodied abelisaurid taxon described from the Lameta Formation of India, in addition to *Lametasaurus indicus*, *Rajasaurus narmadensis*, *Indosaurus matleyi*, and *Indosuchus raptorius*. As has been discussed previously [16], [43], the lack of overlap between elements of the various taxa prevents a firm statement on the validity of individual taxa. Nonetheless, given the typical diversity of abelisaurids in other formations (one or two species at most), it seems highly unlikely that five large theropods co-existed. The lack of vertical striations on the paradental plates is unique among abelisauroids, and differs from the condition in other abelisaurid premaxillae from the same formation (AMNH 1753; American Museum of Natural History, New York, New York, USA). This undoubtedly contributes to the recovery of *R. gujaratensis* at a position outside Abelisauridae for some trees in the analysis. Although an astragalus is known for *R. gujaratensis*, its anatomy could not be reliably interpreted in the absence of figures, and thus the character related to the anatomy of the ascending process (character 185) is coded as unknown.

*Kryptops palaios* was scored only for the holotype maxilla (MNN GAD1–1), because only this element out of the assigned material is undoubtedly abelisaurid. We agree with recent authors [44] that the partial postcranial skeleton with vertebrae, ribs, pelvis, and sacrum that was originally referred to *K. palaios* [45] is probably an allosauroid, possibly the carcharodontosaurid *Eocarcharia dinops* known from the same unit.

**Figure 11:**
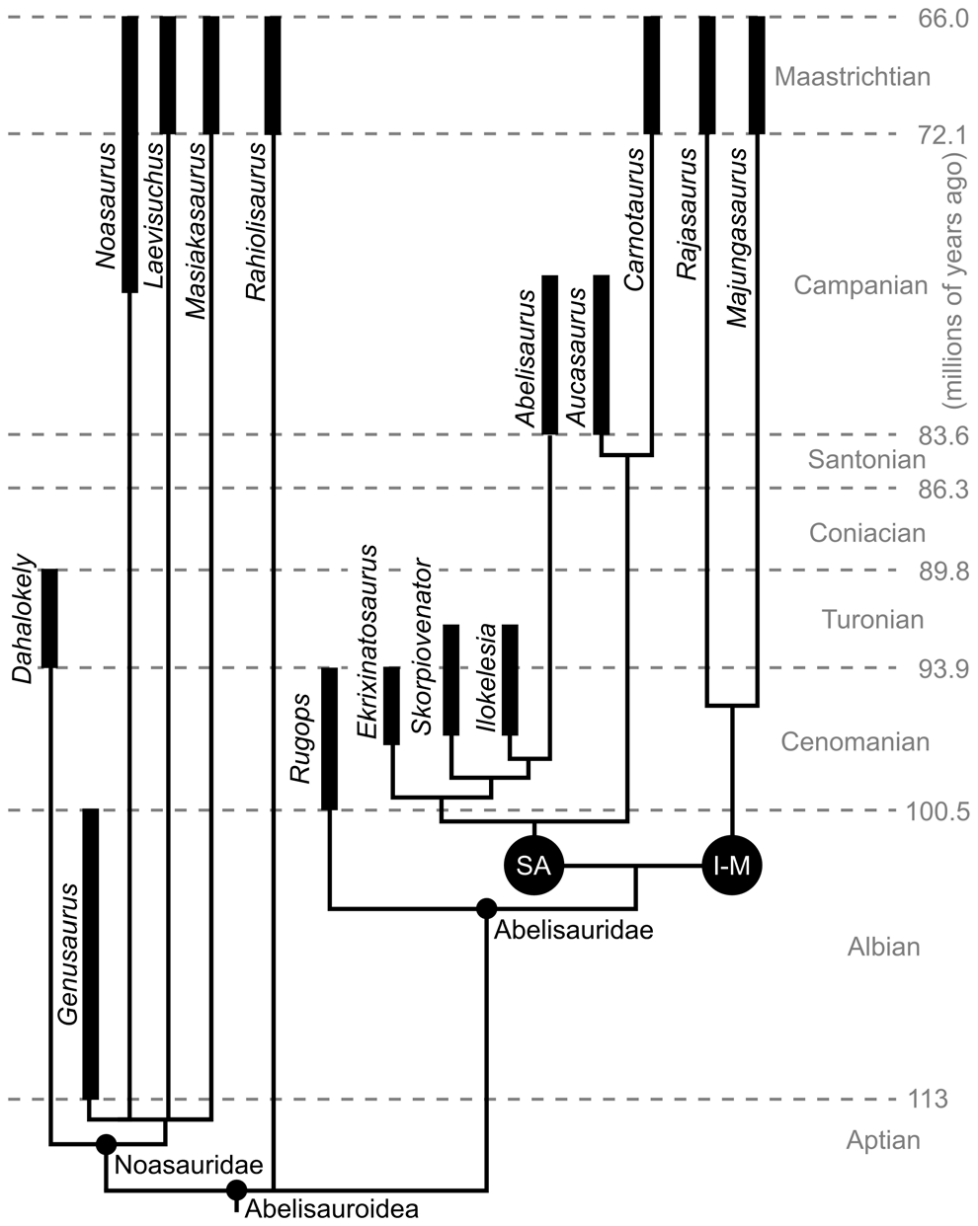
Phylogeny of Abelisauroidea. Topology is based on a reduced strict consensus tree from the phylogenetic analysis, with non-abelisaurids removed (see Figure S6 for full tree). The black bars for each taxon represent uncertainty in age, rather than known duration. The node labeled “I-M” indicates the clade of abelisaurids exclusive to Indo-Madagascar, whereas its sister clade “SA” is exclusive to South America.

Safe taxonomic reduction was implemented using TAXEQ3 [46], identifying *Indosaurus* and *Kryptops* as equivalent to other species, and thus these taxa were removed from further analysis. Additional exploration of the results showed that the very incomplete *Velocisaurus* was highly unstable, leaving resolution only within Abelisauridae but not within the rest of Ceratosauria. Comparisons of trees produced by pruning *Velocisaurus* pre- and post-analysis showed no difference in topology, and so the taxon was also removed from the analysis. The matrix (Dataset S1) was analyzed using TNT 1.1 [47], and the settings for the tree search were as follows: *Herrerasaurus* as the outgroup, Wagner trees as the starting tree, 1,000 replicates, and the TBR (tree bisection reconnection) swapping algorithm, saving 1,000 trees per replication. Branches with a minimum length of zero were collapsed. Decay indices and bootstrap values (with 1,000 replicates, sampling with replacement) were also calculated in TNT (Figure S1). The resulting strict consensus tree with associated branch supports and other statistics is provided in Figure S1, and a tree showing just abelisauroids is presented in Figure 11. A comprehensive list of synapomorphies for each node are presented in Text S1.

A total of 18 equally parsimonious trees of 334 steps were recovered in the phylogenetic analysis (after safe taxonomic reduction removing *Indosaurus* and *Kryptops*, as well as the removal of *Velocisaurus*; consistency index = 0.614; retention index = 0.759; Figures 11, S6). All major, relevant clades (Ceratosauria, Abelisauroidea, Abelisauridae, and Noasauridae) were resolved, although complete resolution at the node Ceratosauria was prevented by the uncertain position of *Berberosaurus liassicus*. When the latter was removed, all major clades of ceratosaurs were resolved, differing only in the resolution of the “elaphrosaur” and noasaurid clades as well as the placement of *Rahiolisaurus*. In all trees, *Dahalokely* was recovered as a noasaurid, supported by two unambiguous synapomorphies (width/height ratio of mid-cervical centra between 2 and 3; centrum in vertebrae D1 and D2 dorsoventrally compressed; Text S1). Two additional steps were required to pull *Dahalokely* outside of Abelisauroidea, and five additional steps were necessary to force *Dahalokely* as immediate sister to *Masiakasaurus* and *Laevisuchus* (Indo-Malagasy noasaurids). However, only one additional step was required to force *Daholokely* into the clade including the Indo-Malagasy abelisaurids *Majungasaurus* and *Rajasaurus*. Thus, the precise position of *Dahalokely* within Abelisauroidea is considered tentative and may change given additional data.

Previous analysis [17] found weak support for *Eoabelisaurus* as an abelisaurid. Here, the taxon is unambiguously recovered as a non-abelisauroid ceratosaur, sister to a node-based Abelisauroidea (i.e., least inclusive clade including Abelisauridae and Noasauridae). An additional two steps were required to force *Eoabelisaurus* as an abelisaurid in this data set.

### Body size estimation

In order to estimate the maximum body length of *Dahalokely tokana* and compare it with other abelisauroids, a set of measurements for three relatively complete abelisauroid taxa were compiled. These include *Carnotaurus sastrei* (MACN-CH 894; Museo Argentino de Ciencias Naturales, Chubut Collection, Buenos Aires, Argentina; measurements from [21]), *Majungasaurus crenatissimus* (UA 8678; University of Antananarivo, Antananarivo, Madagascar; measurements from [22]), and *Masiakasaurus knopfleri* (FMNH PR 2481; Field Museum of Natural History, Chicago, Illinois, USA; measurements from [18]). Estimated body lengths were taken from the literature [21] or extrapolated from published skeletal reconstructions [18], [22]. For *M. crenatissimus*, a reconstruction ([22]:figure 1; based in part on UA 8678) was scaled to the vertebral dimensions of UA 8678, producing an estimated total body length for this specimen of 3.9 m. We note that this individual is smaller than most published body length estimates for *M. crenatissimus* (e.g., 6–7 m long in [48]), but UA 8678 was chosen for the analysis here because it is the most complete axial column known.

The maximum length, cranial width, and cranial height of the vertebral centra were recorded (although width and height on the caudal surface were used for UA 8678). To estimate overall vertebral size, the geometric mean of the measurements was calculated for each vertebra. The ratio of the geometric mean for each vertebral position relative to the equivalent in *D. tokana* was then calculated. This ratio was multiplied by the estimated body length for each taxon to determine an estimated body length for *D. tokana*, and the mean value for these measurements was then calculated. Sample size was too small to develop an adequate regression model for body size, so these estimates should be considered tentative. Measurements and a comprehensive list of body length estimates are included in Text S1.

Relative to *Majungasaurus* (EBL [estimated body length] = 3.9 m), equivalent vertebrae of *Dahalokely* are 67–86% smaller; for *Carnotaurus* (EBL = 9.4 m), 40–46% smaller; for *Masiakasaurus* (EBL = 2.2 m), 190–253% larger. Assuming proportions similar to abelisaurids, this suggests that the holotype for *Dahalokely* was around 3.5 m in total body length (estimated range = 2.6–4.4 m); noasaurid proportions result in an estimated length between 4.2–5.6 m. Although the phylogenetic analysis weakly recovers *Dahalokely* as a noasaurid, the proportions of the cervical vertebra centrum are more similar to those of abelisaurids. Thus, we hypothesize that *Dahalokely* had a relatively shorter neck than seen in noasaurids (Figure 2A), and the shorter overall body length estimates are favored.

## Discussion

Although the holotype material is limited to the axial skeleton, a suite of autapomorphies support the validity of *Dahalokely tokana*. Furthermore, *Dahalokely* can be confidently constrained to the clade including *Eoabelisaurus* and Abelisauroidea, based on two unambiguous synapomorphies (including the broad development of the pre- and postspinal fossae and breadth of the centrum in cervical vertebrae; Text S1). However, the incomplete nature of the material, a mixture of characteristics found in both noasaurids and abelisaurids, and the lack of overlapping elements for some important taxa renders a confident placement of *Dahalokely* within abelisauroids difficult.

*Dahalokely* is the oldest abelisauroid known from Madagascar (Turonian; ∼93.5–89.8 million years ago), preceding *Majungasaurus* and *Masiakasaurus* by at least 20 million years. Undescribed, indeterminate abelisauroid material from the Ankazamihaboka Beds can only be age-constrained to between 88 and 70 million years old [15], [49], and thus provides little additional information beyond establishing the presence of abelisauroids prior to the Maastrichtian. Furthermore, *Dahalokely* is the only known diagnostic dinosaur material from Indo-Madagascar dating to the temporal interval between isolation of the conjoined land mass and their separation.

Although the age and geographic location of *Dahalokely* suggest that it could be ancestral to later Indo-Malagasy taxa, this is not strongly supported in the current phylogenetic analysis. Nonetheless, we hypothesize that this tentative result may be overturned by future work. Undoubtedly, additional fossils from Madagascar and India will do much to clarify the origins of their Cretaceous faunal assemblages.

## Supporting Information

Figure S1 ***Dahalokely tokana*** **holotype (UA 9855), interactive figure of ?fifth cervical (C?5) vertebra reconstructed from CT scan data.**
(PDF)

Figure S2 ***Dahalokely tokana*** **holotype (UA 9855), interactive figure of ?first dorsal (D?1) vertebra reconstructed from CT scan data.**
(PDF)

Figure S3 ***Dahalokely tokana*** **holotype (UA 9855), interactive figure of ?second dorsal (D?2) vertebra reconstructed from CT scan data.**
(PDF)

Figure S4 ***Dahalokely tokana*** **holotype (UA 9855), interactive figure of ?sixth dorsal (D?6) vertebra reconstructed from CT scan data.**
(PDF)

Figure S5 ***Dahalokely tokana*** **holotype (UA 9855), interactive figure of ?eighth dorsal (D?8) vertebra reconstructed from CT scan data.**
(PDF)

Figure S6 **Phylogeny of Ceratosauria, strict consensus tree. Numbers above each node indicate Bremer support nodes (decay indices); numbers below each node indicate bootstrap values (only given for values above 25%).**
(TIF)

Text S1 **Supplementary text, including differential diagnoses, full character list for phylogenetic analysis, TNT format data matrix for phylogenetic analysis, list of unambiguous synapomorphies, and comparative measurements for vertebrae.**
(DOC)

Dataset S1 **Character matrix for phylogenetic analysis of Ceratosauria in TNT format.**
(TNT)
