# Supplementary figures and images for: An Abelisauroid Theropod Dinosaur from the Turonian of Madagascar
Source: PLoS One. 2013 Apr 18;8(4):e62047. doi: 10.1371/journal.pone.0062047 (PMC3630149; doi:10.1371/journal.pone.0062047)

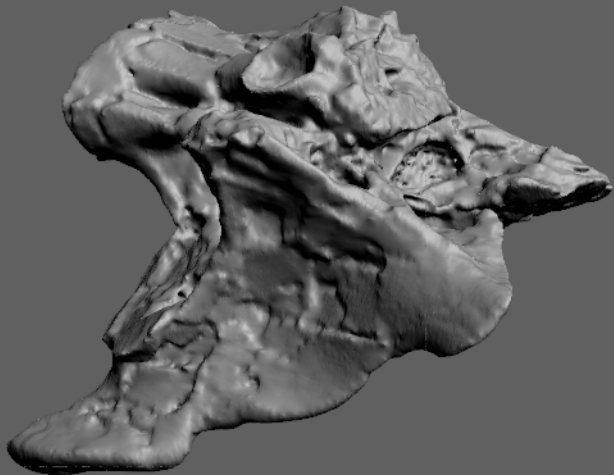

Supplement: Figure S1 — Dahalokely tokana holotype (UA 9855), interactive figure of ?fifth cervical (C?5) vertebra reconstructed from CT scan data. (PDF) [file pone.0062047.s001.pdf]

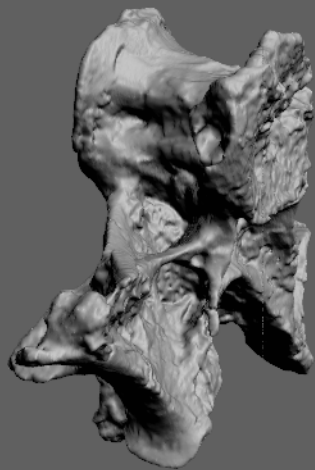

Supplement: Figure S2 — Dahalokely tokana holotype (UA 9855), interactive figure of ?first dorsal (D?1) vertebra reconstructed from CT scan data. (PDF) [file pone.0062047.s002.pdf]

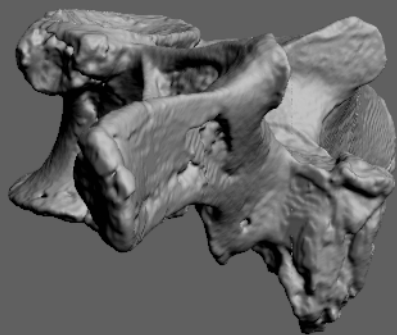

Supplement: Figure S3 — Dahalokely tokana holotype (UA 9855), interactive figure of ?second dorsal (D?2) vertebra reconstructed from CT scan data. (PDF) [file pone.0062047.s003.pdf]

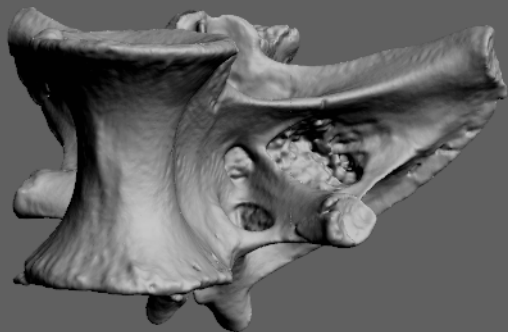

Supplement: Figure S4 — Dahalokely tokana holotype (UA 9855), interactive figure of ?sixth dorsal (D?6) vertebra reconstructed from CT scan data. (PDF) [file pone.0062047.s004.pdf]

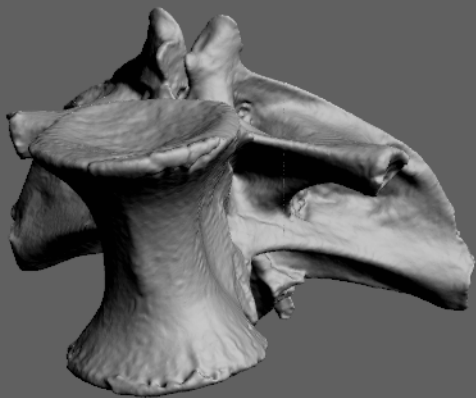

Supplement: Figure S5 — Dahalokely tokana holotype (UA 9855), interactive figure of ?eighth dorsal (D?8) vertebra reconstructed from CT scan data. (PDF) [file pone.0062047.s005.pdf]

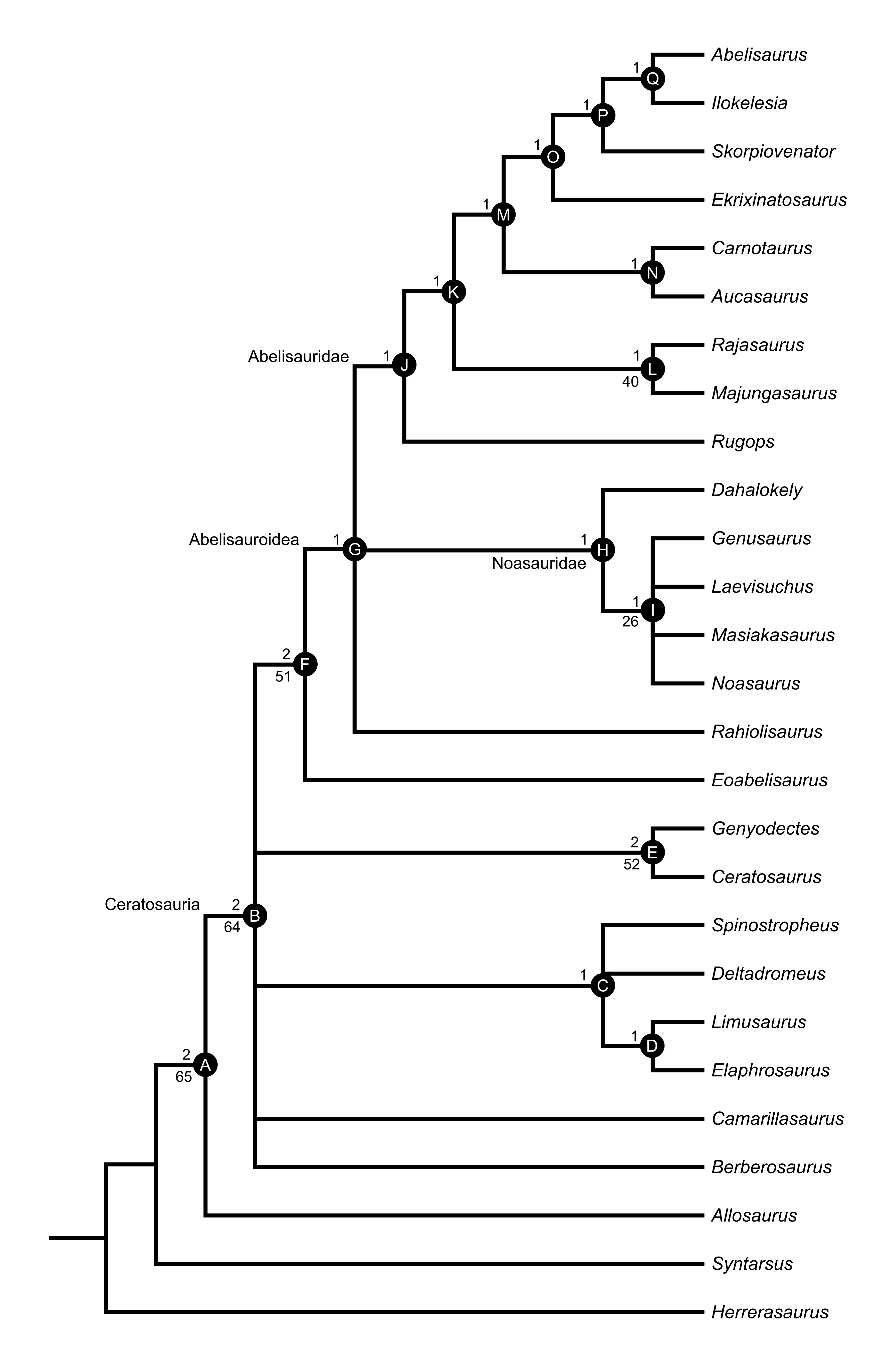

Supplement: Figure S6 — Phylogeny of Ceratosauria, strict consensus tree. Numbers above each node indicate Bremer support nodes (decay indices); numbers below each node indicate bootstrap values (only given for values above 25%). (TIF) [file pone.0062047.s006.tif]
