# Supplementary material for: An Abelisauroid Theropod Dinosaur from the Turonian of Madagascar
Source: PLoS One. 2013 Apr 18;8(4):e62047. doi: 10.1371/journal.pone.0062047 (PMC3630149; doi:10.1371/journal.pone.0062047)
Supplement: Text S1 — Supplementary text, including differential diagnoses, full character list for phylogenetic analysis, TNT format data matrix for phylogenetic analysis, list of unambiguous synapomorphies, and comparative measurements for vertebrae. (DOC) [file pone.0062047.s007.doc]

**Supplementary Text S1**

**A Non-Avian Theropod Dinosaur from the Turonian of Madagascar**

**Andrew A. Farke and Joseph J. W. Sertich**

The following additional information is contained in this file:

S1.1. CHARACTERS FOR PHYLOGENETIC ANALYSIS

S1.2. TNT FORMAT DATA MATRIX FOR PHYLOGENETIC ANALYSIS

S1.3. SYNAPOMORPHIES FOR NODES ON STRICT CONSENSUS TREE

S1.4. ESTIMATION OF BODY LENGTH FOR *DAHALOKELY TOKANA*

**S1.1. CHARACTERS FOR PHYLOGENETIC ANALYSIS**

The backbone for the phylogenetic analysis was the matrix of Carrano and Sampson (2008), and the wording and conceptualization for all characters is taken from that work unless otherwise indicated. Five characters are novel to this analysis, and the remainder were taken from other recently published analyses (Canale et al., 2009; Pol and Rauhut, 2012). These characters are described below.

**Skull**

1. *External surface of maxilla and nasal: smooth (0), sculptured (1).* Character 1 of Carrano and Sampson (2008).
2. *External surface of postorbital, lacrimal and jugal: smooth (0), sculptured (1).* Character 2 of Carrano and Sampson (2008).
3. *Maxillary process of premaxilla: well-developed (0), reduced to a short triangle (1).* Character 3 of Carrano and Sampson (2008).
4. *Subnarial foramen: enclosed (0), reduced/open dorsally (1).* Character 4 of Carrano and Sampson (2008).
5. *Height/length ratio of premaxilla ventral to external naris: 0.5–2.0 (0), > 2.0 (1).* Character 5 of Carrano and Sampson (2008).
6. *Proportions/presence of the anterior ramus of the maxilla: absent (0), anteroposteriorly long (1), or tall and blunt (2).* Character 6 of Carrano and Sampson (2008).
7. *Facet for nasal articulation on maxilla: shallow, anterolateral (0), socket, lateral (1).* Character 7 of Carrano and Sampson (2008).
8. *Palatal process of maxilla: long and ridged (0), short and rectangular (1).* Character 8 of Carrano and Sampson (2008).
9. *Anteroventral border of antorbital fenestra: graded or stepped (0), demarcated by raised ridge (1).* Character 9 of Carrano and Sampson (2008).
10. *Ventral portion of antorbital fossa: present on maxilla (0), absent (1).* Character 10 of Carrano and Sampson (2008).
11. *Anteroposterior length of maxillary–jugal contact relative to total maxilla length: less than 40% (0), more than 40% (1).* Character 11 of Carrano and Sampson (2008).
12. *Nasal–nasal contact in adults: separate (0), partly or fully fused (1).* Character 12 of Carrano and Sampson (2008).
13. *Row of foramina on dorsal nasal surface: absent (0), present (1).* Character 13 of Carrano and Sampson (2008).
14. *Posterior narial margin: fossa (0), laterally splayed hood (1).* Character 14 of Carrano and Sampson (2008).
15. *Location of nasal–frontal contact relative to highest point of orbit: anterior (0), directly above (1).* Character 15 of Carrano and Sampson (2008).
16. *Condition of prefrontal in adults: separate (0), partly or completely fused (1).* Character 16 of Carrano and Sampson (2008).
17. *Frontals: unfused (0), or fused (1).* Character 37 of Canale et al., 2009.
18. *Frontal–parietal contact in adults: separate (0), fused at least on the dorsal surface (1).* Character 17 of Carrano and Sampson (2008), modified following Character 22 of Pol and Rauhut (2012).
19. *Skull roof dorsoventral thickness: thin, relatively flat (0), thickened (1).* Character 18 of Carrano and Sampson (2008).
20. *Skull roof ornamentation: none (0), midline (1), lateral (2).* Character 19 of Carrano and Sampson (2008).
21. *Arrangement of bones along dorsal margin of orbit: postorbital and lacrimal separated by frontal, which forms part of orbital rim (0), contact between postorbital and lacrimal that excludes frontal from orbital rim (1).* Character 20 of Carrano and Sampson (2008).
22. *Knob-like dorsal projection of parietals and supraoccipital: absent (0), present (1).* Character 21 of Carrano and Sampson (2008).
23. *Development of median parietal skull table: flat, broad (0), narrow, with sagittal crest (1).* Character 22 of Carrano and Sampson (2008).
24. *Size and elevation of nuchal wedge and parietal alae: moderate (0), tall and expanded (1).* Character 23 of Carrano and Sampson (2008).
25. *Size of infratemporal fenestra: subequal to size of orbit or smaller (0), considerably enlarged, larger than orbit and especially expanded ventrally (1).* Modified from character 38 of Rauhut (2003); character 30 of Pol and Rauhut (2012).
26. *Postorbital suborbital flange: absent (0), present (1).* Character 24 of Carrano and Sampson (2008).
27. *Anteroposterior length of postorbital relative to height: markedly less (0), equal to or greater (1).* Character 25 of Carrano and Sampson (2008).
28. *Length of the posterior process of the postorbital: less than half of the length of the anterior process (0); more than half, but less than the length of the anterior process (1); longer than the anterior process (2).* Character 33 of Pol and Rauhut (2012); modified from character 47 of Canale et al. (2009). ORDERED.
29. *Orientation of posterior edge of postorbital: vertical (0), sloped anteroventrally (1).* Character 26 of Carrano and Sampson (2008).
30. *Morphology of anteroventral portion of ventral process of the postorbital: confluent with remainder of process (0), step and fossa present (1).* Character 27 of Carrano and Sampson (2008).
31. *Jugal process of the postorbital: with distinct kink in posterior margin at the beginning of the jugal facet (0), jugal facet continuous with posterior margin of the dorsal part of the postorbital (1).* Character 35 of Pol and Rauhut (2012).
32. *Posterior margin of the ventral process of the postorbital: convex in at least its dorsal part (0); straight or slightly concave over its entire length (1).* Character 37 of Pol and Rauhut (2012); modified modified from character 50 of Canale et al. (2009).
33. *Lateral depression on the posterodorsal part of the junction of the anterior, posterior, and ventral postorbital processes: present (0), absent (1).* Wording modified from character 39 of Pol and Rauhut (2012).
34. *Morphology of dorsalmost postorbital–squamosal contact: smooth (0), knob (1).* Character 28 of Carrano and Sampson (2008).
35. *Appearance of postorbital–squamosal contact in lateral view: contact edges visible (0), edges covered by dermal expansions (1).* Character 29 of Carrano and Sampson (2008).
36. *Anterior process of lacrimal: includes antorbital fossa and rim (0), antorbital fossa only (1).* Character 30 of Carrano and Sampson (2008).
37. *Lacrimal fossa: exposed laterally (0), covered by dermal ossifications (1).* Character 31 of Carrano and Sampson (2008).
38. *Suborbital process of lacrimal: absent (0), present (1).* Character 32 of Carrano and Sampson (2008).
39. *Morphology of lacrimal along dorsal orbit rim: flat (0), raised brow or shelf (1).* Character 33 of Carrano and Sampson (2008).
40. *Morphology of jugal–maxilla contact: slot or groove (0), lateral shelf (1).* Character 34 of Carrano and Sampson (2008).
41. *Morphology of jugal–lacrimal articulation: simple butt joint (0), overlapping and pocketed (1).* Character 35 of Carrano and Sampson (2008).
42. *Ventral margin of the posterior half of the jugal: straight or only slightly convex (0); strongly convex (1).* Character 53 of Pol and Rauhut (2012); modified from character 33 of Canale et al. (2009).
43. *Relative lengths of posterior jugal prongs: upper prong much shorter than lower (0), both prongs subequal in length (1).* Character 36 of Carrano and Sampson (2008).
44. *Squamosal contribution to nuchal crest: absent or minimal (0), present and broad (1).* Character 37 of Carrano and Sampson (2008).
45. *Quadrate flange of squamosal: wraps around quadrate head (0), ends posterior to quadrate head (1).* Character 38 of Carrano and Sampson (2008).
46. *Dorsoventral proportions of quadratojugal prongs for jugal: narrow (0), deep (1).* Character 39 of Carrano and Sampson (2008).
47. *Overlap of quadratojugal onto quadrate posteriorly: absent (0), present (1).* Character 40 of Carrano and Sampson (2008).
48. *Quadrate foramen: present (0), absent (1).* Character 41 of Carrano and Sampson (2008).
49. *Ossification of interorbital region: weak or absent (0), extensive (1).* Character 42 of Carrano and Sampson (2008).
50. *Morphology of trigeminal foramen: single (0), partly or fully split (1).* Character 43 of Carrano and Sampson (2008).
51. *Vagal canal opening: through otoccipital (0), onto occiput (1).* Character 44 of Carrano and Sampson (2008).
52. *Depth of basisphenoid recess: shallow (0), deep (1).* Character 45 of Carrano and Sampson (2008).
53. *Shape of opening for basisphenoid recess: ovoid (0), teardrop-shaped (1).* Character 46 of Carrano and Sampson (2008).
54. *Depth of indentation between basal tubera and basisphenoid processes: deep notch (0), shallow embayment (1).* Character 47 of Carrano and Sampson (2008).
55. *Medial fossa ventral to occipital condyle: absent (0), present (1).* Character 48 of Carrano and Sampson (2008).
56. *Size of dorsal groove on occipital condyle: wide (0), narrow (1).* Character 49 of Carrano and Sampson (2008).
57. *Orientation of basioccipital–basisphenoid suture: oblique (0), horizontal (1).* Character 50 of Carrano and Sampson (2008).
58. *Depth of median ridge on supraoccipital: less than width (0), greater than width (1).* Character 51 of Carrano and Sampson (2008).
59. *Morphology of jugal process of palatine: tapered process, triradiate palatine (0), expanded process, tetraradiate palatine (1).* Character 52 of Carrano and Sampson (2008).
60. *Pocket on ectopterygoid flange of the pterygoid: absent (0), present (1).* Character 53 of Carrano and Sampson (2008).
61. *Shape of pterygoid articulation with basipterygoid process: tab-like (0), acuminate (1).* Character 54 of Carrano and Sampson (2008).
62. *Arrangement of jugal and pterygoid processes of ectopterygoid: oblique (0), parallel (1).* Character 55 of Carrano and Sampson (2008).
63. *Proportions of ectopterygoid: gracile (0), robust (1).* Character 56 of Carrano and Sampson (2008).
64. *Ventral excavation into ectopterygoid: absent (0), fossa (1), groove (2).* Character 57 of Carrano and Sampson (2008).
65. *Size of external mandibular fenestra: small to moderate (0), large (1).* Character 58 of Carrano and Sampson (2008).
66. *Position of anterior end of external mandibular fenestra relative to last dentary tooth: posterior (0), ventral (1).* Character 59 of Carrano and Sampson (2008).
67. *Horizontal ridge on lateral surface of surangular below mandibular joint: weak or moderate (0), strong (1).* Character 60 of Carrano and Sampson (2008).
68. *Contour of posterior edge of splenial: straight (0), curved or notched (1).* Character 61 of Carrano and Sampson (2008).
69. *Prongs at anterior end of splenial: one (0), two (1).* Character 62 of Carrano and Sampson (2008).
70. *Morphology of dentary–surangular articulation just above external mandibular fenestra: small notch (0), large socket (1).* Character 63 of Carrano and Sampson (2008).
71. *Shape of articulated dentary rami in dorsal view: V-shaped (0), U-shaped (1).* Character 64 of Carrano and Sampson (2008).
72. *Ventral margin of the dentary: has slight concavity posteriorly (0), or deeply convex along its entire length (1).* Modified from Character 22 of Canale et al. (2009) and character 84 of Pol and Rauhut (2012).
73. *Position of lateral dentary groove: at or above mid-depth (0), in ventral half (1).* Character 65 of Carrano and Sampson (2008).
74. *Position of posterior end of posteroventral process of dentary relative to posterior end of posterodorsal process: far posterior (0), directly ventral (1).* Character 66 of Carrano and Sampson (2008).
75. *Arrangement of premaxillary tooth carinae: nearly symmetrical, on opposite sides (0), more asymmetrical, both on lingual side (1).* Character 67 of Carrano and Sampson (2008).
76. *Number of maxillary teeth: more than 12 (0), 12 or fewer (1).* Character 68 of Carrano and Sampson (2008).
77. *Tooth curvature: substantially-curved mesial and distal profiles with apex positioned distal to distal profile (0), or apex centrally positioned; mesial profile exhibits strong curvature; distal profile is straight or very slightly curved (1)*. Modified from character 5 of Canale et al. (2009) and equivalent to character 89 of Pol and Rauhut (2012). A third character state (from Canale et al., 2008) was removed because it was not applicable to the taxa included in this analysis.
78. *Pronounced size difference between premaxillary and maxillary teeth: absent (0), present (1).* Character 93 of Pol and Rauhut (2012).
79. *Transverse flattening of lateral maxillary teeth: moderate (0), extreme (1).* Character 94 of Pol and Rauhut (2012).
80. *Size of largest maxillary tooth: less (0) or more (1) than height of the dentary.* Character 95 of Pol and Rauhut (2012).
81. *Surface texture of paradental plates: smooth (0), vertically striated or ridged (1).* Character 69 of Carrano and Sampson (2008).
82. *Visibility of paradental plates in medial view: widely exposed (0), obscured (1).* Character 70 of Carrano and Sampson (2008).
83. *Medial groove in paradental plates exposing replacement teeth: present (0), absent (1).* Character 71 of Carrano and Sampson (2008).

**Axial Skeleton**

1. *Neural arch pneumaticity: moderate (0), extreme (1).* Character 72 of Carrano and Sampson (2008).
2. *Internal structure of presacral vertebrae: solid (0), camerate (1), camellate (2).* Character 73 of Carrano and Sampson (2008).
3. *Atlantal epipophysis: short and triangular in lateral outline (0); strongly elongate and rod-like (1).* Character 98 of Pol and Rauhut (2012); modified from character 58 of Canale et al. (2009).
4. *Length of axial epipophyses: moderate (0), long (1).* Character 74 of Carrano and Sampson (2008).
5. *Morphology of axial spinopostzygapophyseal lamina: weakly concave (0), deeply invaginated (1).* Character 75 of Carrano and Sampson (2008).
6. *Development of axial diapophyses: weak, nubbin (0), prominent, pendant (1).* Character 76 of Carrano and Sampson (2008).
7. *Axial pleurocoels: absent (0), present (1).* Character 77 of Carrano and Sampson (2008).
8. *Posterior pleurocoel in postaxial presacral vertebrae: absent (0), fossa only (1), fossa with pneumatic foramen (2).* Character 78 of Carrano and Sampson (2008).
9. *Demarcation of dorsal surface of neural arch from diapophyseal surface in anterior cervical vertebrae: gently sloping (0), ridge (1).* Character 79 of Carrano and Sampson (2008).
10. *Anteroposterior position of cervical neural spines: posterior half of centrum (0), anterior half of centrum (1).* Character 80 of Carrano and Sampson (2008).
11. *Ventral keel on anterior cervicals: present (0), faint or absent (1).* Character 81 of Carrano and Sampson (2008).
12. *Anterior prongs on postaxial cervical epipophyses: absent (0), present (1).* Character 82 of Carrano and Sampson (2008).
13. *Development of pre- and postspinal fossae in postaxial cervical vertebrae: narrow (0), broad (1).* Character 83 of Carrano and Sampson (2008).
14. *Position of cervical zygapophyses: close to midline (0), placed far laterally (1).* Character 84 of Carrano and Sampson (2008).
15. *Morphology of anterior cervical epipophyses: low, blunt (0), long, thin (1), long, robust (2).* Character 85 of Carrano and Sampson (2008).
16. *Length/height ratio of mid-cervical centra: less than 2(0); between 2 and 3 (1), more than 3 (2).* Character 115 of Pol and Rauhut (2012); modified from character 86 of Carrano and Sampson (2008). Ordered.
17. *Ratio between anterior width and height of middle and posterior cervical centra: less (0) or more (1) than 1.3.* Character 107 of Pol and Rauhut (2012).
18. *Height of postaxial cervical neural spines: moderate or tall (0), short (1).* Character 87 of Carrano and Sampson (2008).
19. *Accessory fossa on dorsal surface of postaxial cervical transverse processes: present (0), absent (1).* Character 88 of Carrano and Sampson (2008).
20. *Shape of postaxial cervical zygapophyses: craniocaudally elongated or ovate (0); mediolaterally elongated with lateral half greatly expanded craniocaudally (1).* In *Majungasaurus*, some other abelisaurids, and *Laevisuchus*, the post-axial cervical zygapophyses are longest in the medio-lateral direction, and the lateral halves are expanded craniocaudally (O’Connor, 2007). In other theropods, the zygapophyses are ovate and/or elongated cranio-caudally. New character.
21. *Postzygapophysis in mid-cervical vertebrae: placed at least partially over the posterior end of the neural arch pedicle and does not overhang the centrum posteriorly (0); posterior to the neural arch pedicle and overhangs the centrum posteriorly (1).* Character 112 of Pol and Rauhut (2012); modified from character 54 of Canale et al. (2009).
22. *Attachment of caudal centrodiapophyseal lamina in mid-cervical vertebrae: joins centrum caudal to mid-length (0); joins centrum cranial to mid-length (1).* In *Ilokelesia*, *Masiakasaurus*, and *Dahalokely*, the caudal centrodiapophyseal lamina joins the centrum caudal to its mid-length in mid-cervical vertebrae. In other taxa, such as *Coelophysis*, *Majungasaurus* and *Carnotaurus*, the lamina joins the centrum at or slightly ahead of its mid-point. New character.
23. *Postzygodiapophyseal lamina in mid-cervical vertebrae: present, and connects the diapophysis with the postzygapophysis (0); reduced, indicated by a low ridge in parts (1).* Character 117 of Pol and Rauhut (2012); modified from character 57 of Canale et al. (2009).
24. *Shape of dorsal transverse processes in dorsal view: rectangular (0), triangular (1).* Character 89 of Carrano and Sampson (2008).
25. *Height of dorsal parapophyses: slightly elevated from centrum (0), project far laterally (1).* Character 90 of Carrano and Sampson (2008).
26. *Paradiapophyseal lamina: absent, weak (0), pronounced (1).* Character 91 of Carrano and Sampson (2008).
27. *Dorsal vertebral centrum length ratio relative to height: more than 2 (0), less than 2 (1).* Character 92 of Carrano and Sampson (2008).
28. *Shape of articular surface of centrum in vertebrae D1 and D2: subcircular (0); dorsoventrally compressed (1).* In most theropods and some abelisaurids (e.g., *Carnotaurus*, *Majungasaurus*), the articular surfaces of the vertebra centra for vertebrae D1 and D2 are approximately equal in width and height (e.g., width:height ratio = 1.04 for D2 in *Carnotaurus sastrei* and 0.88 in *Majungasaurus crenatissimus*). In *Dahalokely*, the cranial dorsal vertebrae are strongly dorsoventrally compressed (width:height ratio = 1.53); a similar state is seen in *Rajasaurus*. The vertebra from the holotype of *Rajasaurus narmadensis* previously identified as a mid-cervical centrum (Wilson et al., 2003) is here re-identified as a cranial dorsal (probably D2), because the parapophyses are more dorsally located than seen in other abelisaurid cervical vertebrae. This aspect of the morphology is in close agreement with the D2 vertebra of *Dahalokely*. The ratio in *Spinostropheus* is 1.23 and 1.18 for D1 and D2, respectively, and 1.4 for *Masiakasaurus knopfleri*. New character.
29. *Orientation of prezygapophyses in caudal half of dorsal vertebral series in cranial view: dorsal or dorsomedial (0); dorsolateral (1).* As noted by O’Connor (2007), most theropods have prezygapophyses directed dorsally or dorsomedially in the caudal half of the dorsal vertebral series (when viewed in cranial view). This contrasts with the condition in many abelisauroids (e.g., *Carnotaurus*, *Majungasaurus*, *Dahalokely*, *Masiakasaurus*), in which the prezygapophyses are directed dorsolaterally. New character.
30. *Infradiapophyseal fossa of middle and caudal dorsal vertebrae: undivided (0); divided by caudal paradisophyseal lamina (1)*. In most theropods, the infradiapophyseal fossa is a single cavity on the ventral surface of the transverse process. In abelisauroids, however, a caudal paradiapophyseal lamina on the mid- and caudal dorsal vertebrae (D6-D10 in *Majungasaurus crenatissimus*; O’Connor 2007) divides this fossa into a dorsal and ventral portion. Although previously noted only for abelisaurids (O’Connor, 2007), this feature also occurs in *Masiakasaurus* (e.g., FMNH PR2621), and thus probably characterizes a more inclusive clade. New character.
31. *Number of sacral vertebrae: 2 [primordial sacrals only] (0), 5 [1 dorsosacral, 2 caudosacrals] (1), 6 [2 dorsosacrals, 2 caudosacrals] (2).* Character 93 of Carrano and Sampson (2008).
32. *Transverse dimensions of mid-sacral centra relative to other sacrals: equivalent (0), constricted (1).* Character 94 of Carrano and Sampson (2008).
33. *Orientation of ventral margin of mid-sacral centra: horizontal (0), arched (1).* Character 95 of Carrano and Sampson (2008).
34. *Dorsal edge of sacral neural spines: as thin as remainder of spine (0), thickened (1).* Character 96 of Carrano and Sampson (2008).
35. *Condition of sacral neural spines in adults: separate (0), fused (1).* Character 97 of Carrano and Sampson (2008).
36. *Pneumaticity of sacral neural spines: weak or absent (0), well developed (1).* Character 98 of Carrano and Sampson (2008).
37. *Morphology of anterior caudal neural spines: sheet-like (0), rod-like (1).* Character 99 of Carrano and Sampson (2008).
38. *Longitudinal ventral groove in anterior caudal vertebrae: present (0), absent (1).* Character 130 of Pol and Rauhut (2012); modified from character 120 in Rauhut (2003).
39. *Centrodiapophyseal laminae in anterior mid-caudal vertebrae: absent (0); present as low, rounded ridges (1).* Character 131 of Pol and Rauhut (2012).
40. *Proportions of anterior caudal neural arch base relative to mid-centrum proportions: smaller (0), equal or greater (1).* Character 100 of Carrano and Sampson (2008); modified following character 132 of Pol and Rauhut (2012).
41. *Distal morphology of anterior to mid-caudal transverse processes: tapering (0), posteriorly expanded (1).* Modified from character 101 of Carrano and Sampson (2008); character 134 of Pol and Rauhut (2012).
42. *Anterior process at distal end of anterior to mid-caudal transverse processes: absent (0), present (1).* Modified from character 101 of Carrano and Sampson (2008); character 135 of Pol and Rauhut (2012).
43. *Neural spines of mid-caudals: inclined posteriorly (0); rectangular and sheet-like (1); anteroposteriorly short and vertical (2).* Character 136 of Pol and Rauhut (2012); Character 124 of Rauhut (2003).
44. *Proximodistal length of anterior caudal transverse processes: less (0), or more (1) than 1.4 times the length of caudal centra.* Character 137 of Pol and Rauhut (2012).
45. *Contact between cervical vertebrae 1 cervical ribs in adults: separate (0), fused (1).* Character 102 of Carrano and Sampson (2008).
46. *Wing-like process at the base of the anterior cervical rib shafts: absent (0), present (1).* Character 103 of Carrano and Sampson (2008).
47. *Bifurcate cervical rib shafts: absent (0), present (1).* Character 104 of Carrano and Sampson (2008).

**Appendicular skeleton**

1. *Relative width of scapular blade: broad, more than twice glenoid depth (0), narrow, less than twice glenoid depth (1).* Character 105 of Carrano and Sampson (2008).
2. *Large vertical depression on lateral side of scapula above the glenoid: absent (0), present (1).* Character 141 of Pol and Rauhut (2012).
3. *Distal expansion of scapula: present (0), absent (1).* Character 142 of Pol and Rauhut (2012).
4. *Development of posteroventral process on coracoid: moderate (0), pronounced (1).* Character 106 of Carrano and Sampson (2008).
5. *Spacing between glenoid and posteroventral process of coracoid: moderate (0), close (1).* Character 107 of Carrano and Sampson (2008).
6. *Size of coracoid: shallow (0), very deep (1).* Character 108 of Carrano and Sampson (2008).
7. *Shape of humeral head: elongate (0), globular (1).* Character 109 of Carrano and Sampson (2008).
8. *Shape of distal humeral condyles: rounded (0), flattened (1).* Character 110 of Carrano and Sampson (2008).
9. *Placement of humeral greater tubercle relative to internal tuberosity: proximal (0), distal (1).* Character 111 of Carrano and Sampson (2008).
10. *Longitudinal torsion of humeral shaft: absent (0), present (1).* Character 112 of Carrano and Sampson (2008).
11. *Size of deltopectoral crest: prominent (0), low (1).* Character 113 of Carrano and Sampson (2008).
12. *Shape of humerus in lateral view: S-shaped (0), straight (1).* Character 150 of Pol and Rauhut (2012); Character 143 of Rauhut (2003).
13. *Length of humerus relative to femur length, more than one-third (0), less than one-third (1).* Character 114 of Carrano and Sampson (2008).
14. *Length of radius: more (0) or equal to or less than half the length of the humerus (1).* Character 145 of Rauhut (2003); character 154 of Pol and Rauhut (2012); approximately equivalent to character 88 of Canale et al. (2009).
15. *Distal articular surface of radius: flat or slightly concave (0); strongly convex (1).* Character 155 of Pol and Rauhut (2012); modified from character 90 of Canale et al. (2009).
16. *Ventrolateral expansion of lateral distal condyle of Mc II: present (0), absent (1) (new character).* Character 157 of Pol and Rauhut (2012).
17. *Metacarpal IV: slender, width of mid-shaft less than half of the width of mid-shaft of MC II (0), robust, width more than half of that of MC II (1).* Character 158 of Pol and Rauhut (2012).
18. *Constricted neck between articular ends of manual phalanges: present (0), absent (1).* Character 160 of Pol and Rauhut (2012).
19. *Relative length of longest manual phalanges: more than twice width (0), less than twice width (1).* Character 115 of Carrano and Sampson (2008).
20. *Development and curvature of manual unguals: well developed and highly recurved with distal end ventrally deflected forming an angle of at least 90 degrees with respect to the proximal articular surface (0), or absent or reduced in size and straight or poorly curved (1).* Character 162 of Pol and Rauhut (2012).
21. *Contacts between pelvic elements in adults: separate (0), fused (1).* Character 116 of Carrano and Sampson (2008).
22. *Posterior width of iliac brevis fossa: subequal to anterior width (0), twice anterior width (1).* Character 117 of Carrano and Sampson (2008).
23. *Morphology of lateral ilium between supra-acetabular crest and brevis shelf: gap (0), continuous (1).* Character 118 of Carrano and Sampson (2008).
24. *Anterior margin of ilium: faces anteriorly or anterodorsally (0); faces anteroventrally (1).* Character 167 of Pol and Rauhut (2012).
25. *Shape of posterior margin of iliac postacetabular process: convex (0), undulating (1).* Character 119 of Carrano and Sampson (2008).
26. *Shape of dorsal margin of iliac postacetabular process: convex (0), straight (1).* Character 120 of Carrano and Sampson (2008).
27. *Relative sizes of iliac–pubic and iliac–ischial articulations: subequal (0), iliac–pubic articulation larger (1).* Character 121 of Carrano and Sampson (2008).
28. *Relative proximodistal length of pubic and ischial peduncle: pubic peduncle considerably longer than ischial peduncle (0); peduncle of subequal length, or ischial peduncle longer (1).* Character 171 of Pol and Rauhut (2012).
29. *Orientation of ischial peduncle of ilium: posteroventrally inclined (0); vertical (1).* Character 172 of Pol and Rauhut (2012).
30. *Shape of anterior margin of iliac preacetabular process: rounded (0), undulating (1).* Character 122 of Carrano and Sampson (2008).
31. *Anteroventral lobe of iliac preacetabular process: absent (0), present (1).* Character 123 of Carrano and Sampson (2008).
32. *Contact between pubic apices: separate (0), contacting (1).* Character 124 of Carrano and Sampson (2008).
33. *Morphology of contact between pubis and ilium: planar (0), peg-and-socket (1).* Character 125 of Carrano and Sampson (2008).
34. *Morphology of dorsal surface of pubic boot on midline: convex (0), concave (1).* Character 126 of Carrano and Sampson (2008).
35. *Notch ventral to obturator process on ischium: absent (0), present (1).* Character 127 of Carrano and Sampson (2008).
36. *Morphology of distal ischium: rounded, separate (0), expanded, triangular, fused (1).* Character 128 of Carrano and Sampson (2008).
37. *Morphology of contact between ischium and ilium: planar (0), peg-and-socket (1).* Character 129 of Carrano and Sampson (2008).
38. *Proportions of limb bones: moderate to gracile (0), robust (1).* Character 130 of Carrano and Sampson (2008).
39. *Dimorphism in hind limb morphology: absent (0), present (1).* Character 131 of Carrano and Sampson (2008).
40. *Ratio of length of femur to length of ilium: more than 1.3 (0), less than 1.1 (1).* Character 164 of Pol and Rauhut (2012); modified from character 82 of Canale et al. (2009).
41. *Morphology of anterolateral muscle attachments on proximal femur: continuous trochanteric shelf (0), distinct lesser trochanter and attachment bulge (1).* Character 132 of Carrano and Sampson (2008).
42. *Development of medial epicondyle of femur: rounded (0), ridge (1), long flange (2).* Character 133 of Carrano and Sampson (2008).
43. *Morphology and orientation of femoral tibiofibularis crest: narrow, longitudinal (0), broad, oblique (1).* Character 134 of Carrano and Sampson (2008).
44. *Distal expansion of tibial cnemial process: absent (0), present (1).* Character 135 of Carrano and Sampson (2008).
45. *Shape of distal tibia in distal view: rounded (0), mediolaterally elongate (1).* Character 136 of Carrano and Sampson (2008).
46. *Anterior side of distal end of tibia: with marked step, demarcating the border of the ascending process of the astragalus (0), flat (1).* Character 190 of Pol and Rauhut (2012); character 207 of Rauhut (2003).
47. *Development of fibular fossa on medial aspect of proximal fibula: posterior groove (0), posteriorly open fossa (1), medially open fossa (2).* Character 137 of Carrano and Sampson (2008).
48. *Size of iliofibularis tubercle on fibula: moderate (0), large (1).* Character 138 of Carrano and Sampson (2008).
49. *Contact between fibula and ascending process of astragalus in adults: separate (0), fused (1).* Character 139 of Carrano and Sampson (2008).
50. *Morphology of astragalar ascending process: blocky (0), laminar (1).* Character 140 of Carrano and Sampson (2008).
51. *Orientation of astragalar distal condyles: ventral (0), 10–30◦ anterior (1), 30–45◦ anterior (2).* Character 141 of Carrano and Sampson (2008).
52. *Horizontal groove across the anterior face of the astragalar condyles: absent or weak (0), pronounced (1).* Character 142 of Carrano and Sampson (2008).
53. *Contact between astragalus and calcaneum in adults: separate (0), fused (1).* Character 143 of Carrano and Sampson (2008).
54. *Development of astragalar articular surface for distal end of fibula: large, dorsal (0), reduced, lateral (1).* Character 144 of Carrano and Sampson (2008).
55. *Height of the ascending process of the astragalus relative to depth of astragalar body: less or equal (0), greater (1).* Character 145 of Carrano and Sampson (2008).
56. *Width of metatarsal II relative to widths of metatarsals III and IV: subequal (0), reduced (1).* Character 146 of Carrano and Sampson (2008).
57. *Proportions of distal end of metatarsal IV: broader than tall (0), taller than broad (1).* Character 147 of Carrano and Sampson (2008). Partially overlapping with character 202 of Pol and Rauhut (2012) and character 112 of Canale et al. (2009).
58. *Antarctometatarsus: absent (0), present (1).* Character 148 of Carrano and Sampson (2008).
59. *Morphology of lateral and medial grooves on pedal unguals: single (0), double (1).* Character 149 of Carrano and Sampson (2008).
60. *Mediolateral symmetry of pedal digit II ungual: symmetrical (0), asymmetrical (1).* Character 150 of Carrano and Sampson (2008).
61. *Length of pedal digit phalanges I-1 + I-2 relative to III-1: greater (0), less than or equal (1).* Character 151 of Carrano and Sampson (2008).
62. *Ventral surface of pedal unguals with a well developed flexor tubercle, and usually flat (0), or without flexor tubercle and with a ventral depression in its proximal end (1).* Character 205 of Pol and Rauhut (2012); character 113 of Canale et al. (2009).

**S1.2. TNT FORMAT DATA FILE FOR PHYLOGENETIC ANALYSIS**

**Note:** Because TNT numbers characters starting at “0”, a dummy character (all coded as “?”) has been added as character 0. This ensures that the characters match up with the numbering in the character list.

xread 193 30

Herrerasaurus ?00000000010000000000000000010000[0 1]00000000000?000000000000000000010000000000000010000000000000000001000?1?00001??00000000?00000000010100000000000010000000000000000000000000000000000000000000000

Syntarsus ?00000100100000000000000000010000000000000?000000000000100000000100000000000000000000100000100000012010?1?11000??0100010000000001000000100000000000000?110011010000000000100110000000001000010000

Allosaurus ?0000010000000000000010000002000000000000000000000111011000110101001100000010000000001001110001001001000000000100010000000000000000100000000100000000000000001000110010000[0 1]1000102001210110000010

Abelisaurus ?1111121?01?101111102111111011011011?111?1?1?111?10???10011??1???1??1????????????101?????????????????????????????????????????????????????????????????????????????????????????????????????????????

Aucasaurus ?11?1121?011??1??11?2?????0?????????110?1???????????????????????????????????01??????????????1????????1????????????[1 2]?????1?1111?1???0??10111111111111?1??11?11???111???1?0??1?111??1?1111??0011111

Berberosaurus ????????????????????????????????????????????????????????????????????????????????????11?????200??00?0010?00[0 1]????????11?????????????????????????????0?????????????????????01?11??1011??????????????

Camarillasaurus ????????????????????????????????????????????????????????????????????????????????????0???????????00??0?????????1???211????1?0??0????1??101?????????????????????????????????????1??????????????????

Carnotaurus ?1111121?011011011112111111101110111110111111110111???100111?111?111111111110100010112?1111210111120110101011110112111111111?1?10110111011111111111111?111111111111?11110?1111???11????1???0?????

Ceratosaurus ?00001210000001000000001010020010000000000000000?11?110000111011100111100011001110011210111200100120000?0?011010?1210?1?000000201000010000111111001001?111??01000?111111011[0 1]111101101111100010???

Dahalokely ????????????????????????????????????????????????????????????????????????????????????12?1???2?0?011?111010001111111???????????????????????????????????????????????????????????????????????????????

Deltadromeus ????????????????????????????????????????????????????????????????????????????????????????????????????????????????????????0?????1?????1?101011?01???????????????????????1?0??11001121?111?110110???

Ekrixinatosaurus ?1??1?2??011?????1100?1?1?1?0??????1????????1????1??????????????????????11??01????0?1??????21?0011?011?10?1???1???[1 2]???0?0???11??0??????????????????????1???111??111?????0??11?110???1??1?00?????1

Elaphrosaurus ????????????????????????????????????????????????????????????????????????????????????1[1 2]?????1001000?2111?1?11100???21011?0100??1?1??01?1110111110??10?0?1111101000110?0100?010?01121?1101??011????

Eoabelisaurus ??0????????????000100000110020111000?10?????01?011?????00?1??????????????????0?0?0??1[1 2]?????210?011?011010001111???21111?111110200??011?0000??[0 1]1010111111111?0100?11?011???1[0 1][0 1]11101101111??0?011?1

Genusaurus ??????????????????????????????????????????????????????????????????????????????????????????????????????????????0???[1 2]????????????????????????????????????1?11?1111?1??????0??12?1??11??????????????

Genyodectes ?0???0?????????????????????????????????????????????????????????????????000?[0 1]?0111001?????????????????????????????????????????????????????????????????????????????????????????????????????????????

Ilokelesia ??1???????????????????????1111111001????????????????1???????????????????????????????1??????2101011200101001?111?11????????1?112?011??????????????????????????????????????????????????????????11?1

Indosaurus ???????????????11?1111111????????????????????????11?????1????????????????????????????????????????????????????????????????????????????????????????????????????????????????????????????????????????

Kryptops ?11?1?21001??????????????????????????????????????????????????????????????????1???101?????????????????????????????????????????????????????????????????????????????????????????????????????????????

Laevisuchus ????????????????????????????????????????????????????????????????????????????????????1???????11?001?1?10110????0?????????????????0????????????????????????????????????????????????????????????????

Limusaurus ?000?0?0???0?0100??0000??000?001??000000000???00?????????????????1-0????0000--???-??1??0100?0010??02?1?????1??0?????????0?[0 1]000?010001111100101101?1[0 1]111?1100110000??001?0?01??????1?11???11???01?

Majungasaurus ?11111210011101111111111111101010101111011111111111111101111111121111111011101000101121111121010112011010101111011[1 2]1111110110021011011101111111?1111111?1111111011?11??11??1??11011111111100111?1

Masiakasaurus ?01?1?21110????0000000????0000010100?001??????????11111001???????10?1110000110?0?01112?111121110011211001001110111[1 2]011110011000?01101?1011111110????11?111?11110??11111101112111111111111111111?0

Noasaurus ?0??1?21110?????????????????????????????????????1???????????????????????????10??00111[1 2]??????11?1011???001?0?????????????????????010???????????????????0?????????????????0?????????????????1??????

Rahiolisaurus ???????????????????????????????????????????????????????????????????????????1?????00?1??1111?10101120??01?1?????????????????1????0?????10???????????????111?11100???1????0??1111?011???11?????????

Rajasaurus ?11????????????1??1111111???????????????1????????1111??01???????211????1?0110????10112?????21?1?????1?0????11111??210?11?1??????0??????????????????????0?0???110???1???11??11111?11?1?????00?????

Rugops ?111112100111110100001110???????????111111?????????????????????????????????101?0?101????????????????????????????????????????????0????????????????????????????????????????????????????????????????

Skorpiovenator ?11?1121?011????1???01???111111101011111?111??11?????????????????1?1????111?010?0???????????1??0112??10?0?1???????????????1?11?1011????????????????????1?11?011011??????0?1111???11?????????????1

Spinostropheus ????????????????????????????????????????????????????????????????????????????????????12?????100100002001000111100?02??111????????000??????01???????????????????????1?????0??????1?????????????????

Velocisaurus ????????????????????????????????????????????????????????????????????????????????????????????????????????????????????????????????????????????????????????????????????????0??????11???11111?111????

;

proc /;

comments 0

;

**S1.3. SYNAPOMORPHIES FOR NODES ON STRICT CONSENSUS TREE**

Unambiguous synapomorphies for nodes on the strict consensus tree were calculated in TNT 1.1 (Goloboff et al., 2008). The letters identifying each node are provided in Figure S6, and the number of the synapomorphic character and its state at the node are provided below.

**Node A:** 28(2), 50(1), 51(1), 52(1), 59(1), 60(1), 62(1), 68(1), 88(1), 94(1), 104(0), 140(1), 157(1), 161(1), 162(1), 171(1), 175(1), 180(1), 182(1), 184(1), 185(1), 191(1)

**Node B (Ceratosauria):** 114(2), 115(1), 121(1), 134(1)

**Node C:** 99(2), 102(1), 106(1), 110(0)

**Node D:** 135(1)

**Node E:** 78(1), 79(1), 80(1)

**Node F:** 92(1), 96(1), 100(1)

**Node G (Abelisauroidia):** 156(1)

**Node H (Noasauridae):** 99(1), 111(1)

**Node I:** 110(0)

**Node J (Abelisauridae):** 81(1)

**Node K:** 17(1), 18(1)

**Node L:** 20(1), 56(1), 168(1)

**Node M:** 30(1), 72(1), 124(1), 125(1)

**Node N:** 20(2), 38(0)

**Node O:** 106(1)

**Node P:** 28(1)

**Node Q:** 32(1), 33(0)

**S1.4. ESTIMATION OF BODY LENGTH FOR *DAHALOKELY TOKANA***

**Table S1.** Comparative measurements of selected vertebrae and body length estimates for various abelisauroid theropods. See main text for explanation of calculations. Abbreviations: BL, body length; DEBL, *Dahalokely* estimated body length; H, height of centrum; L, length of centrum; W, width of centrum. A dash indicates an unpreserved measurement.

| **Position** | **Taxon** | **L (mm)** | **W (mm)** | **H (mm)** | **GM (mm)** | **Ratio** | **BL (m)** | **DEBL (m)** |
| --- | --- | --- | --- | --- | --- | --- | --- | --- |
| **C5** | *Majungasaurus* | 63.2 | 53.3 | 42.8 | 52.4 | 0.67 | 3.9 | 2.6 |
| **C5** | *Carnotaurus* | 119 | 82 | 56 | 81.8 | 0.43 | 9.4 | 4.0 |
| **C6** | *Masiakasaurus* | 32.7 | 16.2 | 11.7 | 18.4 | 1.9 | 2.2 | 4.2 |
| **C5** | *Dahalokely* | 58.7 | 37.6 | 19.4 | 35.0 |  |  |  |
| **D1** | *Majungasaurus* | 59.3 | 53.5 | 60.6 | 57.7 | 0.86 | 3.9 | 3.3 |
| **D1** | *Carnotaurus* | 100 | 114 | − | 106.8 | 0.46 | 9.4 | 4.4 |
| **D1** | *Dahalokely* | 43.5 | 64.5 | 43 | 49.4 |  |  |  |
| **D2** | *Majungasaurus* | 51.9 | 54.3 | 62 | 55.9 | 0.84 | 3.9 | 3.3 |
| **D2** | *Carnotaurus* | 101 | 112 | 108 | 106.9 | 0.44 | 9.4 | 4.1 |
| **D2** | *Dahalokely* | 48.7 | 55.7 | 37.6 | 46.7 |  |  |  |
| **D6** | *Majungasaurus* | 59.7 | 57.9 | 66.6 | 61.3 | 0.76 | 3.9 | 3.0 |
| **D6** | *Carnotaurus* | 117 | 106 | 106 | 109.5 | 0.42 | 9.4 | 4.0 |
| **D6** | *Masiakasaurus* | 28 | 15.3 | 14.4 | 18.3 | 2.53 | 2.2 | 5.6 |
| **D6** | *Dahalokely* | 52.1 | 46.9 | 40.7 | 46.3 |  |  |  |
| **D7** | *Masiakasaurus* | 28.7 | 17.5 | 15.3 | 19.7 | 2.41 | 2.2 | 5.3 |
| **D7** | *Dahalokely* | 55.1 | 44.4 | 44.1 | 47.6 |  |  |  |
| **D8** | *Majungasaurus* | 67.4 | 63.4 | 65.5 | 65.4 | 0.73 | 3.9 | 2.8 |
| **D8** | *Carnotaurus* | 122 | 117 | 117 | 118.6 | 0.4 | 9.4 | 3.8 |
| **D8** | *Dahalokely* | 55.2 | 47.4 | 41.3 | 47.6 |  |  |  |

**ADDITIONAL REFERENCES**

Canale, J., C. Scanferla, F. Agnolin, and F. Novas. 2009. New carnivorous dinosaur from the Late Cretaceous of NW Patagonia and the evolution of abelisaurid theropods. Naturwissenschaften 96:409–414.

Carrano, M. T., and S. D. Sampson. 2008. The phylogeny of Ceratosauria (Dinosauria: Theropoda). Journal of Systematic Palaeontology 6:183–236.

Goloboff, P. A., J. S. Farris, and K. C. Nixon. 2008. TNT, a free program for phylogenetic analysis. Cladistics 24:774–786.

O’Connor, P. M. 2007. The postcranial axial skeleton of *Majungasaurus crenatissimus* (Theropoda: Abelisauridae) from the Late Cretaceous of Madagascar. Society of Vertebrate Paleontology Memoir 8:127–162.

Pol, D., and O. W. M. Rauhut. 2012. A Middle Jurassic abelisaurid from Patagonia and the early diversification of theropod dinosaurs. Proceedings of the Royal Society B: Biological Sciences 279:3170–3175.

Rauhut, O. W. M. 2003. The interrelationships and evolution of basal theropod dinosaurs. Special Papers In Palaeontology 69:1–213.

Wilson, J. A., P. C. Sereno, S. Srivastava, D. K. Bhatt, A. Khosla, and A. Sahni. 2003. A new abelisaurid (Dinosauria, Theropoda) from the Lameta Formation (Cretaceous, Maastrichtian) of India. Contributions from the Museum of Paleontology [University of Michigan] 31:1–42.
